# cZFP609 Tethering BiP Alleviates Cartilage Degradation in Osteoarthritis via Remedying Aberrant ER‐Mitochondrial Contacts

**DOI:** 10.1002/mco2.70405

**Published:** 2025-10-13

**Authors:** Yu Song, Jun‐Long Luo, Fan Zhang, Jie Shi, Shuai Du, Hai‐Bin Jiang, Wen‐Di Zhang, Si‐Ying Chen, Dan‐Dan Zhang, Peng Kong, Yuan Gao, Mei Han, Han Li

**Affiliations:** ^1^ Department of Biochemistry and Molecular Biology College of Basic Medicine Key Laboratory of Neural and Vascular Biology of Ministry of Education Key Laboratory of Vascular Biology of Hebei Province Hebei Medical University Shijiazhuang China; ^2^ Department of Orthopaedic Surgery Institute of Biomechanical Science and Biomechanical Key Laboratory of Hebei Province Third Hospital of Hebei Medical University Shijiazhuang China

**Keywords:** Osteoarthritis, cZFP609, binding immunoglobulin protein, ferroptosis

## Abstract

Vascular dysfunction is implicated in the pathogenesis of osteoarthritis (OA). Herein, we utilized smooth muscle specific human *Sirt1* transgenic (sm*Sirt1*‐Tg) mice characterized by vascular homeostasis to prepare an OA model to validate vasculature‐derived articular cartilage protective factors. The OA of sm*Sirt1*‐Tg mice exhibited significantly reduced cartilage destruction and pain sensitivity, accompanied by increased proteoglycans content and collagen type II (Col2ɑ) expression and decreased matrix metallopeptidase 13 (MMP13) and p53 expression. Vascular smooth muscle cell‐derived cZFP609 was highly enriched in the articular cartilage and plasma of sm*Sirt1*‐Tg mice. Overexpression of cZFP609 abrogated TNFα‐induced endoplasmic reticulum (ER) stress and fine‐tuned the mitochondrial homeostasis in chondrocytes. Mechanistically, cZFP609, located in the cytoplasm, interacted with binding immunoglobulin protein (BiP) to stabilize the BiP oligomeric form. This interaction reduced the level of active BiP monomer that induced not only ER stress via activating IRE1α (inositol‐requiring enzyme 1α) signaling but also mediated the formation of ER–mitochondria contacts (ERMCs). Increased oligomeric BiP by overexpression of cZFP609 suppressed ERMC‐driven aberrant ER–mitochondria communication and diminished lipid peroxidation and ferroptosis, which contributed to maintaining mitochondrial homeostasis and alleviating cartilage degeneration in OA. Taken together, these results elucidate a beneficial cZFP609‐driven feed‐forward circuit that can be effectively targeted to stem the progression of OA.

## Graphical abstract

cZFP609 mediates the cross‐talk between vasculature and cartilage. VSMC‐derived cZFP609 stabilizes the oligomeric form of BiP in chondrocytes, which suppresses TNFα‐induced ER stress signaling and abolishes aberrant ERMC remodeling, and thereby alleviates ferroptosis of chondrocytes and OA progression.

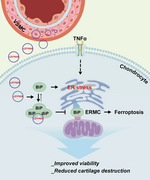

## 1 Introduction

Osteoarthritis (OA) is a multifactorial degenerative disease related to aging, inflammation, overweight, and excessive exercise, which is characterized by progressive pain and cartilage degeneration, accompanied by a significantly increased risk for disability, loss of mobility, and other age‐associated conditions. So far, there are no clinically curative treatments for OA, highlighting the urgent need for a comprehensive understanding of OA pathogenesis and the identification of new targets [1]. Vascular remodeling represents the principal pathophysiological basis of cardiovascular disease (CVD), such as atherosclerosis, restenosis, and hypertension. OA and CVD, both very common diseases, often coexist and share some risk factors such as aging, chronic low‐grade inflammation, obesity, and lifestyle, suggesting the possibility of one interacting with another. Indeed, systemic vascular pathology has already been demonstrated to be implicated in the etiology and pathogenesis of OA [2]. Although multiple evidence suggests a potential effect of vascular disease on OA, data demonstrating how blood vessels affect OA initiation and joint deterioration remain elusive.

The ischemia caused by vascular diseases results in reduced cartilage nutrition and inflicts multiple bone infarcts of advanced OA [3]. Vascular smooth muscle cells (VSMCs) not only maintain vascular physiological function but also contribute to vascular remodeling in varied ways. Sirtuin 1 (SIRT1) protects against stress‐induced vascular remodeling [4, 5, 6]. We have demonstrated that the VSMCs from smooth muscle specific human *Sirt1* transgenic (sm*Sirt1*‐Tg) mice expressed and secreted a noncoding circular RNA (circRNA) cZFP609 via exosomes, which is demonstrated to suppress hypoxia‐induced angiogenesis that is common in OA [7]. These findings prompted us to ask whether and how VSMCs communicate directly with chondrocytes and modulate the structure and function of articular cartilage.

Herein, we demonstrate that sm*Sirt1*‐Tg VSMC‐derived cZFP609 shifts into articular cartilage with no blood vessel and suppressed chondrocyte ferroptosis, associated with ameliorated OA progression.

## 2 Results

### 2.1 OA Is Alleviated in sm*Sirt1*‐Tg Mice

**FIGURE 1:**
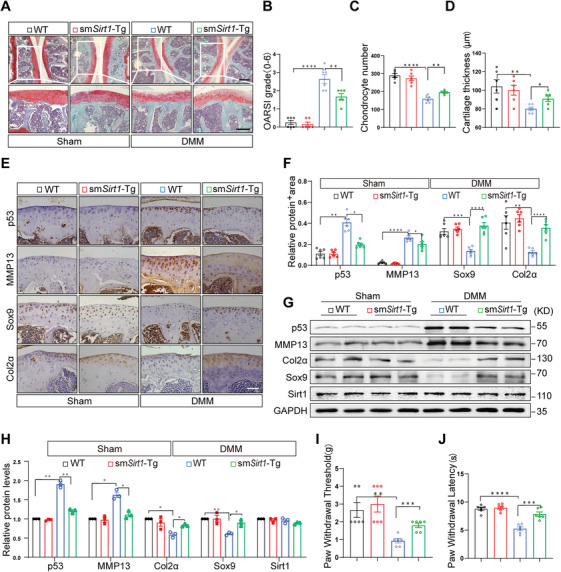
Osteoarthritis is ameliorated in the OA model of sm*Sirt1*‐Tg mice. (A–D) WT and sm*Sirt1*‐Tg mice were induced by DMM surgery (*n* = 6). Safranin‐O/fast green staining (A) and scoring of OA parameters, including OARSI grade (B), chondrocyte number (C), and cartilage thickness (D). Scale bar = 100 µm. (E, F) Immunohistochemical staining for p53, MMP13, Sox9, and Col2ɑ proteins in the cartilage tissue (E) and corresponding quantitative analysis (F). Scale bar = 50 µm. (G, H) Western blot analysis of protein levels of p53, MMP13, Col2ɑ, Sox9, and Sirt1 in the cartilage tissue (G) and corresponding quantitative analysis (H). (I, J) PWT (I) and PWL (J) were used to evaluate mechanical pain sensitivity and hyperalgesia. Data and images represent at least three independent experiments. Statistical analyses, paired *t*‐tests, and Tukey's multiple comparisons tests. *p < 0.05, **p < 0.01, ***p < 0.001, and ****p < 0.0001 versus the corresponding control.

To compare the cartilage degeneration of wild‐type (WT) and sm*Sirt1*‐Tg mice in OA development, we prepared an OA model by destabilization of the medial meniscus (DMM) [8]. OA development of the knee joints was significantly alleviated in 16‐week‐old sm*Sirt1*‐Tg mice, which exhibited significantly increased SO&FG staining of proteoglycans compared to WT mice at 4 weeks after DMM (Figure 1A, Figure S1), accompanied by lower OARSI grades, increased chondrocyte number, and cartilage thickness (Figure 1B–D), indicating an ameliorated cartilage destruction. Immunohistochemistry revealed reduced expression of p53 and MMP13 and increased Sox9 and Col2ɑ expression in the articular cartilage of sm*Sirt1*‐Tg mice compared to WT mice at 4 weeks after surgery (Figure 1E,F). However, the expression of these genes was no different in the sham group between WT and sm*Sirt1*‐Tg mice. Similarly, Western blot showed the p53 and MMP13 expression was lower and Sox9 and Col2ɑ level higher in sm*Sirt1*‐Tg articular cartilage than that in WT control, while SIRT1 expression was no different between WT and sm*Sirt1*‐Tg mice (Figure 1G,H). Further, OA pain at 4 weeks was evaluated by mechanical paw withdrawal threshold (PWT) and thermal paw withdrawal latencies (PWL), respectively. The OA model of WT and sm*Sirt1*‐Tg mice showed markedly reduced values of PWT and PWL compared with the corresponding sham groups. WT mice in the DMM group were more sensitive to mechanical and hot stimuli, and the data of PWT and PWL were lower than that of sm*Sirt1*‐Tg animals (Figure 1I,J), suggesting a mitigation of neuropathic pain behaviors and an increased pain threshold value in sm*Sirt1*‐Tg mice.

### 2.2 Expression of cZFP609 Is Associated With Mitigated Inflammatory Senescence of Chondrocytes

**FIGURE 2:**
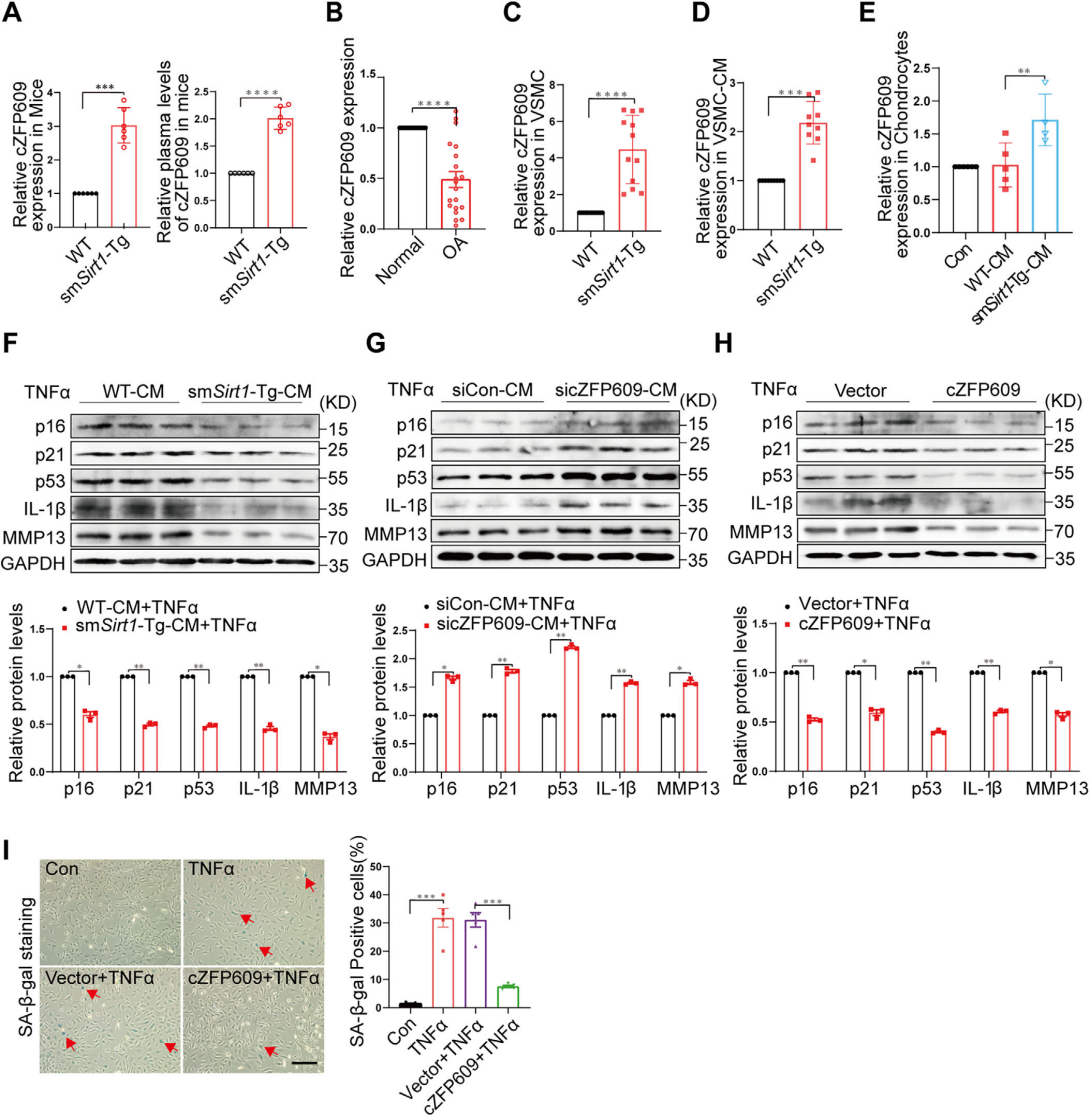
Overexpression of cZFP609 protects chondrocytes from inflammation and degeneration. (A–E) RT‐qPCR of cZFP609 expression. (A) In the articular cartilage (left) and plasma (right) of WT and sm*Sirt1*‐Tg mice (*n* = 6). (B) The plasma level of cZFP609 in OA patients and normal control subjects (*n* = 20). (C) In the VSMCs of WT and sm*Sirt1*‐Tg mice. (D) In WT and sm*Sirt1*‐Tg VSMC conditional media (CM). (E) Chondrocytes were incubated with WT and sm*Sirt1*‐Tg VSMCs CM for 24 h. (F–H) Western blot analysis of p16, p21, p53, IL‐1β, and MMP13 in chondrocytes. (F) Incubated with WT and sm*Sirt1*‐Tg VSMCs CM after treatment with TNFα (20 ng/mL). (G) Incubated with CM from the cZFP609 siRNA‐transfected sm*Sirt1*‐Tg VSMCs. (H) Transfected with vector or cZFP609 plasmid DNA for 12 h and then treated with TNFα (20 ng/mL) for 48 h. (I) SA‐β‐gal staining and levels. Scale bar = 200 µm. Data and images represent at least three independent experiments by paired *t*‐test and Sidak's multiple comparisons test. *p < 0.05, **p < 0.01, ***p < 0.001, and ****p < 0.0001 versus the corresponding control.

Inflammation‐induced chondrocyte senescence is involved in the pathogenesis of OA [9]. Noncoding circRNAs play vital roles in the regulation of gene expression, cellular metabolism, and immune responses [10]. We have demonstrated that cZFP609 is released from VSMCs by exosomes to inhibit hypoxia‐induced angiogenesis [7]. Given that angiogenesis is a key feature in OA, we hypothesized that cZFP609 may be a potential protective factor for OA. We first detected the level of cZFP609 expression in the articular cartilage and plasma of WT and sm*Sirt1*‐Tg mice and showed a higher cZFP609 level in sm*Sirt1*‐Tg samples compared with WT mice (Figure 2A). Intriguingly, the level of plasma cZFP609 was lower in OA patients than that of the normal control subjects (Figure 2B, Table S1). Furthermore, the expression of cZFP609 in VSMCs and their conditional media (CM) was detected. We showed that cZFP609 level was significantly higher in sm*Sirt1*‐Tg VSMCs and corresponding CM than that of WT cells (Figure 2C,D). To determine the source of cZFP609 observed in the articular cartilage, we next examined and showed a lower level of cZFP609 in chondrocytes, which was reversed by sm*Sirt1*‐Tg‐VSMC‐CM incubation (Figure 2E), suggesting that VSMC‐derived cZFP609 is delivered to chondrocytes.

Notably, sm*Sirt1*‐Tg‐CM suppressed TNFα‐induced inflammatory senescence, which exhibited reduced expression of p16, p21, p53, IL‐1β, and MMP13, as indicated by Western blot (Figure 2F). To validate the role of cZFP609 from sm*Sirt1*‐Tg‐CM in inflammatory responses, chondrocytes were incubated with the CM of the cZFP609 specific siRNA‐treated sm*Sirt1*‐Tg VSMCs. As expected, the decrease in expression of these genes was abolished in the cells incubated with the cZFP609 knocked‐down sm*Sirt1*‐Tg‐CM (Figure 2G, Figure S2A,B). To further identify the inhibitory effect of cZFP609 on the inflammatory response of chondrocytes, the cZFP609 expression plasmid was transduced into chondrocytes [7]. We showed that cZFP609 overexpression abrogated TNFα‐induced expression of the inflammation‐related genes in chondrocytes, compared with vehicle control (Figure 2H, Figure S2C). SA‐β‐gal staining showed a suppressed senescence in chondrocytes with overexpression of cZFP609 (Figure 2I). Together, these results suggested that the cZFP609 attenuates the inflammatory senescence of chondrocytes.

### 2.3 Overexpression of cZFP609 Contributes to Mitochondrial Function and Homeostasis of Chondrocytes

**FIGURE 3:**
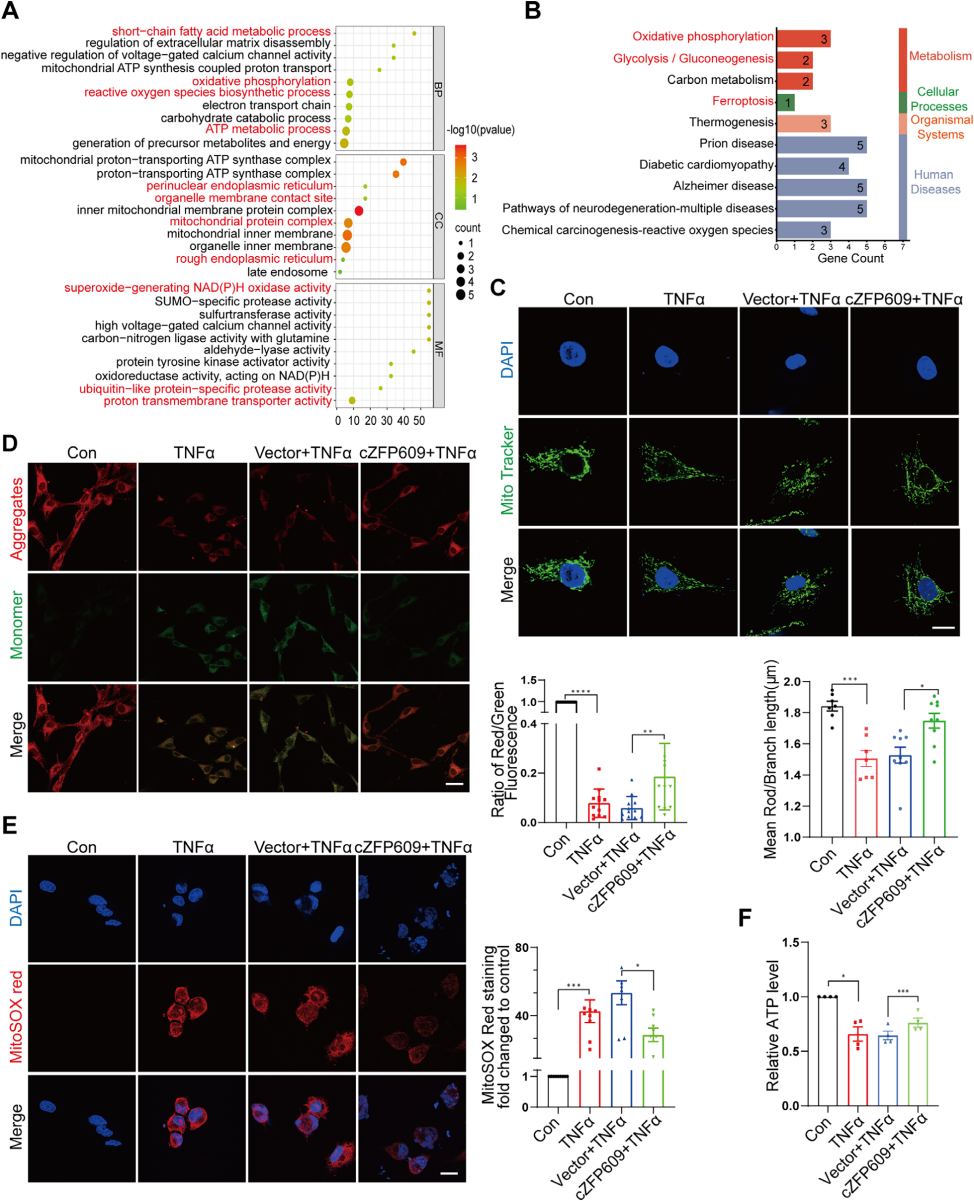
cZFP609 contributes to mitochondrial homeostasis in chondrocytes. (A) Analysis of GO terms enriched by differentially expressed genes in the category of biological process (BP), cellular component (CC), and molecular function (MF) in chondrocytes treated with TNFα (20 ng/mL) after being transfected with vector or cZFP609. (B) Analysis of KEGG pathways enriched by differentially expressed genes of transcriptomics analysis in the above conditions. (C) Representative images and relative quantification by image Fiji macro tool for the mitochondrial morphology of chondrocytes treated with the indicated conditions. Scale bar = 20 µm. (D) The mitochondrial membrane potential was detected with JC‐1 and quantified by the Fiji image macro tool. Scale bar = 40 µm. (E) Mitochondrial ROS (MitoSOX Red) were detected by incubation with MitoSOX and quantified by the Fiji image macro tool. Scale bar = 20 µm. (F) ATP levels were quantified in chondrocytes, the ATP content was calculated as nmol/mg of protein, and the data are represented as the rate of control. Data and images represent at least three independent experiments using the paired *t*‐test and Sidak's multiple comparisons test. *p < 0.05, **p < 0.01, ***p < 0.001, and ****p < 0.0001 versus the corresponding control.

To examine the cZFP609‐mediated pathways involved in OA development in an unbiased manner, we conducted a transcriptomic analysis in cZFP609‐overexpressed chondrocytes after TNFα treatment (Figure S3). Compared with the vehicle group, overexpression of cZFP609 showed 49 downregulated and 46 upregulated pathways in the context of TNFα treatment (Figure S4). GO analysis revealed that differentially expressed genes (DEGs) were enriched in the processes related to oxidative phosphorylation, ROS biosynthesis, and ATP metabolism, and in the cellular components of endoplasmic reticulum and organelle membrane contact sites (Figure 3A). KEGG analysis indicated that the pathways involved in cZFP609 overexpression were associated with ferroptosis, energy metabolism, and degenerative disease (Figure 3B). We discovered that mitochondrial fragmentation was substantially increased in chondrocytes upon TNFα induction (Figure 3C), indicating that longer rods are breaking up into smaller rods or punctate. Increased fragmentation was abolished by overexpression of cZFP609, and the mean rod/branch length markedly increased, presenting classical rod‐like and branched mitochondrial forms in the context of TNFα treatment.

We next examined the effect of cZFP609 on the mitochondrial function. The reduced mitochondrial membrane potential (Δψm) and elevated ROS level were validated by staining using JC‐1 (5,5′,6,6′‐Tetrachloro‐1,1′,3,3′‐tetraethyl‐imidacarbocyanine iodide) and Mito‐SOX in TNFα‐induced chondrocytes (Figure 3D,E). Moreover, TNFα treatment significantly reduced ATP production (Figure 3F), indicating mitochondrial dysfunction. In contrast, overexpression of cZFP609 remedied mitochondrial function, which increased in Δψm and ATP production and reduced in ROS level (Figure 3D–F). These results suggested that cZFP609 was essential for mitochondrial function, especially in maintaining mitochondrial bioenergetics and ROS homeostasis in response to inflammation.

### 2.4 Binding Immunoglobulin Protein Is the Potential Target for cZFP609 in Chondrocytes

**FIGURE 4:**
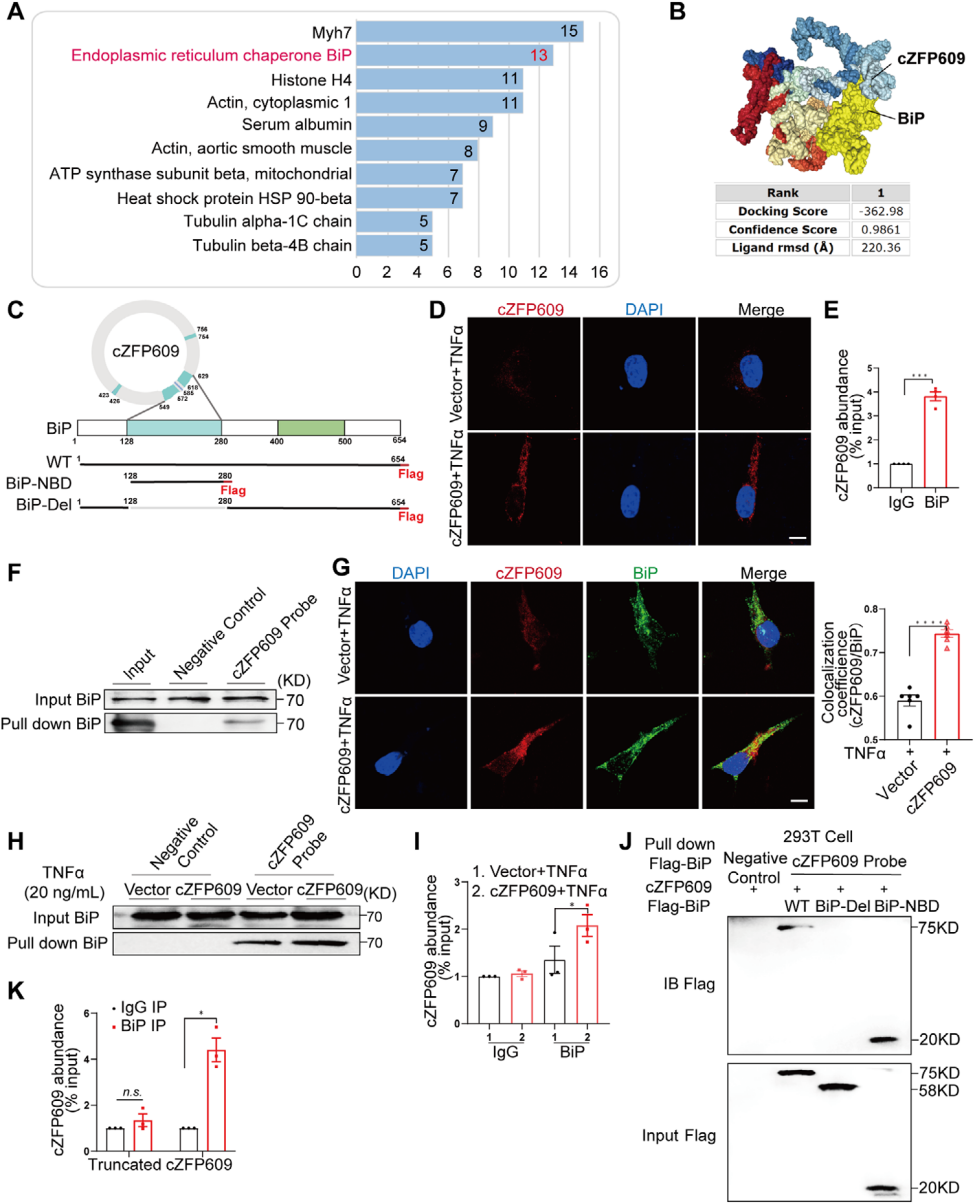
cZFP609 interacts with BiP in chondrocytes. (A) Analysis of cZFP609‐Protein Interactions by mass spectrometry after RNA‐Protein Pull‐down Assays. (B) HDOCK SERVER predicted the binding site of cZFP609 and BiP. (C) Schematic showing cZFP609, full‐length and truncated BiP. (D) Fluorescence in situ hybridization (FISH) assay for the localization of cZFP609 in chondrocytes. Scale bars = 10 µm. (E, F) RNA immunoprecipitation (RIP) assay (E) and RNA pull‐down assay (F) for the interactions between cZFP609 and BiP in chondrocytes. (G) FISH assay for the co‐localization of cZFP609 and BiP in chondrocytes, and quantified by the Fiji image macro tool. Scale bar = 10 µm. (H and I) RNA pull‐down assay (H) and RIP assay (I) for the interactions between cZFP609 and BiP in chondrocytes in the indicated conditions. (J) Representative western blots for mapping the domains of BiP binding to cZFP609. HEK293T cells were transduced with cZFP609 and with either full‐length Flag‐BiP or its truncations. (K) RIP assay for chondrocytes transfected with the cZFP609 full‐length and deletion mutants. Data and images represent at least three independent experiments by a paired *t*‐test. *p < 0.05, ***p < 0.001, and ****p < 0.0001 versus the corresponding control.

To determine the molecular target for cZFP609 maintaining mitochondrial homeostasis, we conducted an RNA pull‐down combined mass spectrometry analysis to identify cZFP609‐bound proteins in cZFP609‐overexpressed primary chondrocytes following TNFα treatment. Among identified proteins (Figure 4A), the ER chaperone binding immunoglobulin protein (BiP) drew most of our attention due to its important role in the regulation of endoplasmic reticulum and mitochondrial functions [11]. We then identified the possible regions of cZFP609 binding to BiP using computational approaches (Figure 4B). The HDOCK SERVER (http://hdock.phys.hust.edu.cn/) predicted two BiP minimal binding regions of cZFP609 as “AAGGGCTCAGAGAAGGCTGCTAAG” (549–572 nt) and “TCATCTAAGGGC” (618–629 nt), and the cZFP609‐binding domain of BiP that overlaps its nucleotide‐binding domain (NBD) (Figure 4C). Using in situ hybridization, we showed that cZFP609 was distributed in the cytoplasm, which significantly increased in the cZFP609‐transfected chondrocytes (Figure 4D). Furthermore, RNA immunoprecipitation (RIP) and RNA pull‐down assays revealed that cZFP609 was also immunoprecipitated by BiP antibody in chondrocytes (Figure 4E), and BiP protein was pulled down by the cZFP609 probe (Figure 4F). In the meantime, the co‐localization of cZFP609 and BiP was further confirmed by fluorescence in situ hybridization (FISH) assay (Figure 4G). This interaction was enhanced by overexpression of cZFP609 (Figure 4G–I), suggesting that BiP is the potential target for cZFP609 in chondrocytes.

To validate the domains that are responsible for the interaction between cZFP609 and BiP, we then constructed two mutants of BiP: flag‐tagged NBD (BiP‐NBD, aa 128–280) that may bind cZFP609 and truncated NBD (BiP‐Del) (Figure 4C), which were transduced into HEK 293T cells. The lysates from BiP full‐length (WT) and BiP‐NBD infected cells were incubated with a biotinylated DNA probe targeting cZFP609. The BiP pulled down by the cZFP609 probe was increased in cells overexpressing flag‐BiP‐NBD and abolished in flag‐BiP‐Del infected cells compared with WT BiP (Figure 4J). Moreover, we constructed a cZFP609‐truncated mutant that deleted the BiP binding regions (549–629 nt) and transduced it into chondrocytes. The lysates from cZFP609 full‐length and its truncated mutant overexpressing chondrocytes were precipitated using a BiP antibody and subjected to reverse transcription‐quantitative PCR (RT‐qPCR) of cZFP609 using specific primers. RIP assay showed that the interaction of cZFP609 with BiP was increased by cZFP609 and diminished by its mutant (Figure 4K). These data suggest that BiP is the target for cZFP609 in chondrocytes.

### 2.5 Overexpression of cZFP609 Inhibits ER Stress Signal Activation via Stabilizing the Oligomeric State of BiP

**FIGURE 5:**
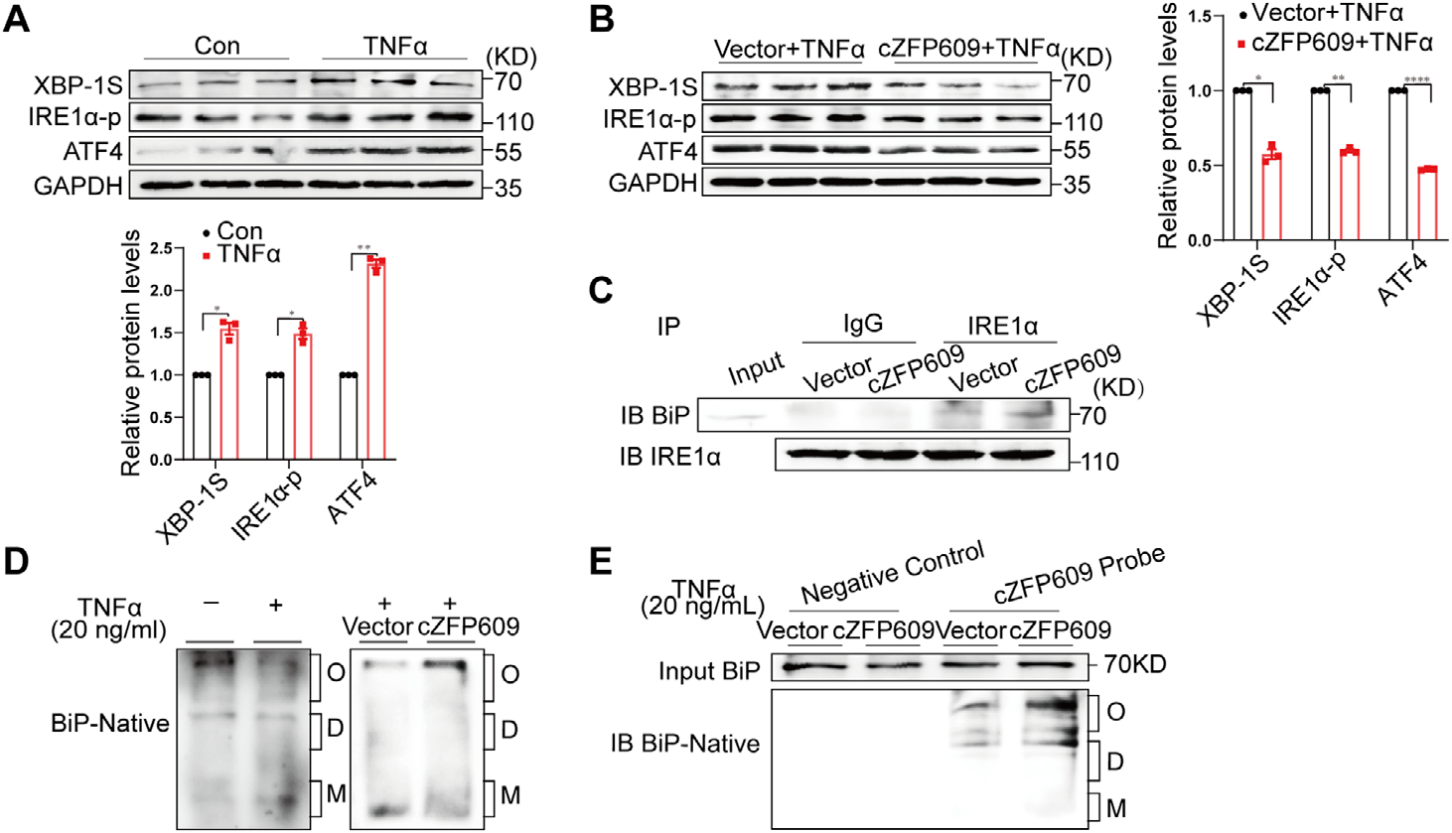
cZFP609 inhibits ER stress via stabilizing oligomeric BiP. (A and B) Western blot analysis of XBP‐1S, IRE1α‐p, and ATF4 proteins in chondrocytes treated with the indicated conditions. (C) Co‐IP assay for the interactions of BiP with IRE1α in chondrocytes. (D) Native PAGE analysis of BiP in chondrocytes. (E) Native PAGE analysis of BiP in chondrocytes after cZFP609 pull‐down assay. Data and images represent at least three independent experiments using Sidak's multiple comparisons test. *p < 0.05, **p < 0.01, and ****p < 0.0001 versus the corresponding control.

BiP localized in the ER lumen is involved in the regulation of protein folding homeostasis and the response to ER stress [12]. To determine the potential significance of cZFP609 binding to BiP in this process, we examined the effect of cZFP609 overexpression on ER stress signalling activity in chondrocytes. Compared to the vehicle, the overexpression of cZFP609 markedly suppressed TNFα‐induced phosphorylation of IRE1α (inositol‐requiring enzyme 1α) and the expression of XBP‐1S and ATF4 (activating transcription factor 4) (Figure 5A,B), indicating decreased ER stress. It has been known that BiP dissociates from the transducers during ER stress, leading to activation of their respective unfolded protein response (UPR) pathways [13]. Notably, the interaction between BiP and IRE1α was enhanced in cZFP609‐overexpressed chondrocytes, consistent with reduced IRE1α phosphorylation (Figure 5C). We speculated that cZFP609 may restrain the dissociation of BiP from IRE1α and thereby reduce ER stress.

Inactive oligomeric BiP can bind the ER sensor IRE1 and PERK to suppress UPR activation [14]. In the presence of stress, BiP converts from inactive oligomeric species into active monomers and dissociates from the sensors, resulting in URP activation [15]. To elucidate the mechanism by which cZFP609 inhibits dissociation of BiP from IRE1α, we performed native PAGE to examine the oligomeric state of BiP in chondrocytes. Under basal conditions, a significant proportion of BiP converts into an inactive oligomeric form (Figure 5D). TNFα treatment significantly decreased oligomeric BiP, accompanied by increased active monomers. Intriguingly, this increase in monomers was abolished by overexpression of cZFP609 and substituted by the oligomer of BiP (Figure 5D). Coincidentally, the RNA pull‐down assay demonstrated that cZFP609‐bound BiP was mainly the oligomer (Figure 5E). These results suggest that cZFP609 maintains the oligomeric state of BiP and thereby inhibits ER stress signal activation.

### 2.6 Overexpression of cZFP609 Attenuates BiP‐Mediated ERMC Remodeling

**FIGURE 6:**
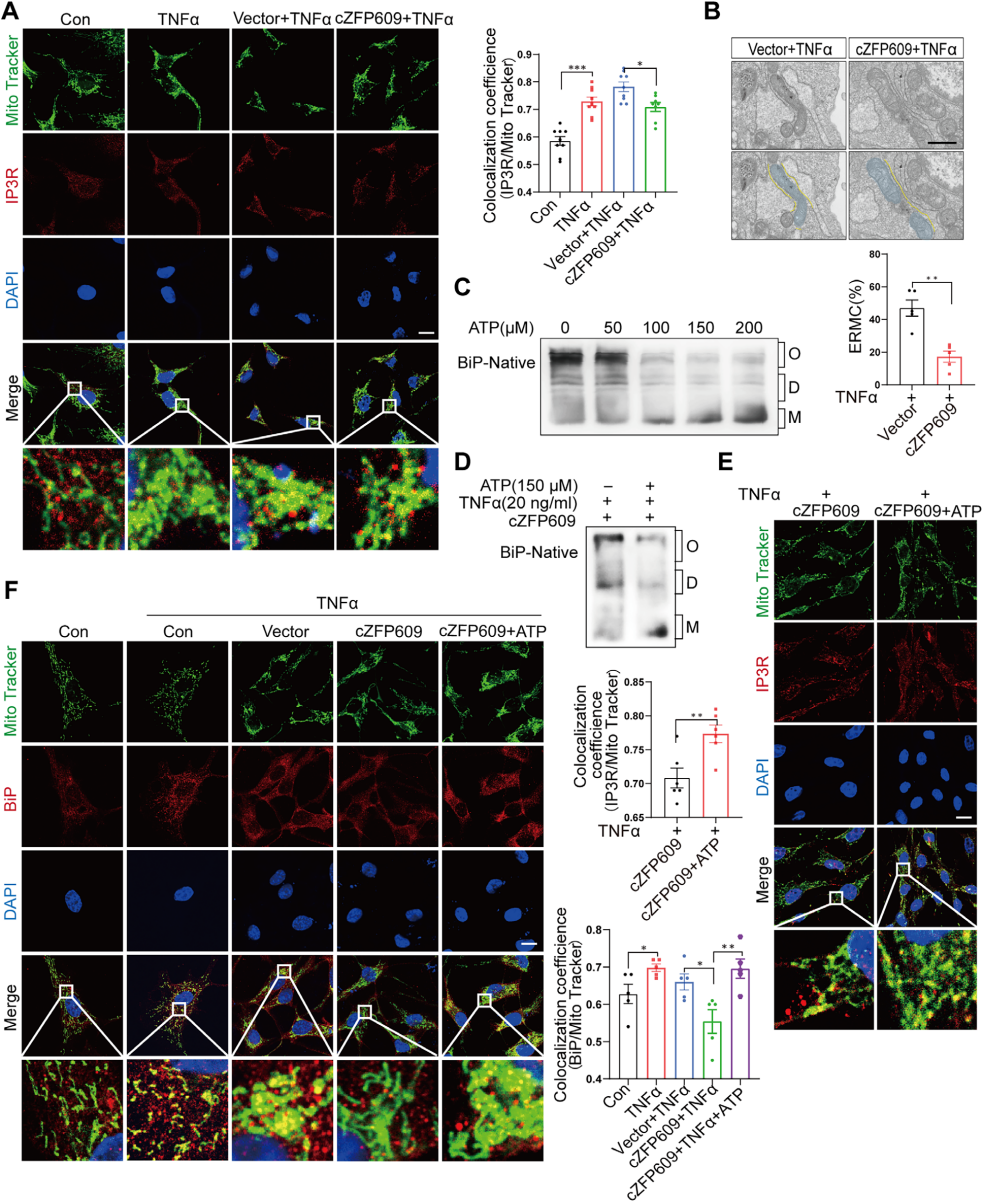
cZFP609 reduces ERMCs via inhibiting the depolymerization of BiP. (A) Representative images of the ERMCs probed with MitoTracker and ER‐resident protein IP3R in chondrocytes treated with the indicated conditions by confocal microscopy and quantified by the Fiji image macro tool. Scale bar = 20 µm. (B) Representative TEM images of ERMCs in chondrocytes treated with the indicated conditions. Mitochondria and ER masks were manually segmented and distinguished in blue and yellow, respectively. ERMCs (distances between 100 and 30 nm) were quantified by the Fiji image macro tool. Scale bar = 500 nm. (C) Native PAGE analysis of BiP in chondrocytes treated with ATP in different concentrations. (D) Native PAGE analysis of BiP in cZFP609 overexpressed chondrocytes exposed to 150 µM ATP after being treated with TNFα (20 ng/mL). (E) Representative images of ERMCs in cZFP609 overexpressed chondrocytes exposed to 150 µM ATP after being treated with TNFα (20 ng/mL), and quantified by the Fiji image macro tool. Scale bar = 20 µm. (F) Representative co‐localization images and relative quantification by image Fiji macro tool in chondrocytes stained with MitoTracker and BiP. Scale bar = 20 µm. Data and images represent at least three independent experiments by paired *t*‐test. *p < 0.05, **p < 0.01, and ***p < 0.001 versus the corresponding control.

To ascertain the relationship between inhibiting ER stress and promoting mitochondrial functions by cZFP609, we monitored the ERMCs in chondrocytes by quantifying IP3R while labeling mitochondria using MitoTracker staining. Quantitative confocal microscopy showed that the co‐localization of both markers was markedly increased upon TNFα treatment and decreased in the chondrocytes simultaneously overexpressing cZFP609 (Figure 6A), supporting that cZFP609 inhibited the ER–mitochondria contacts in the context of inflammation. TEM analysis also confirmed a decreased number of ERMCs (Figure 6B) in cZFP609‐overexpressed chondrocytes.

In the absence of ATP, BiP forms higher‐order oligomers [16]. To verify that reduced ERMCs resulted from increased oligomeric BiP by cZFP609, chondrocytes were treated with ATP to disassemble BiP oligomers. We showed that BiP depolymerization markedly increased in chondrocytes treated with different concentrations of ATP (Figure 6C). Furthermore, cZFP609‐increased oligomeric BiP was abolished by ATP, accompanied by an increase in monomers (Figure 6D). Coincidentally, ERMCs were increased by ATP treatment in cZFP609‐overexpressed chondrocytes (Figure 6E). Importantly, the localization of BiP in the mitochondria was decreased in the context of cZFP609 overexpression, while it was increased in the event of ATP present simultaneously (Figure 6F), suggesting that the active monomers of BiP contributed to ERMC remodeling and cZFP609 reduces ERMCs via inhibiting depolymerization of the BiP.

### 2.7 cZFP609 Suppresses Ferroptosis of Chondrocytes by Inhibiting Depolymerization of BiP In Vitro

**FIGURE 7:**
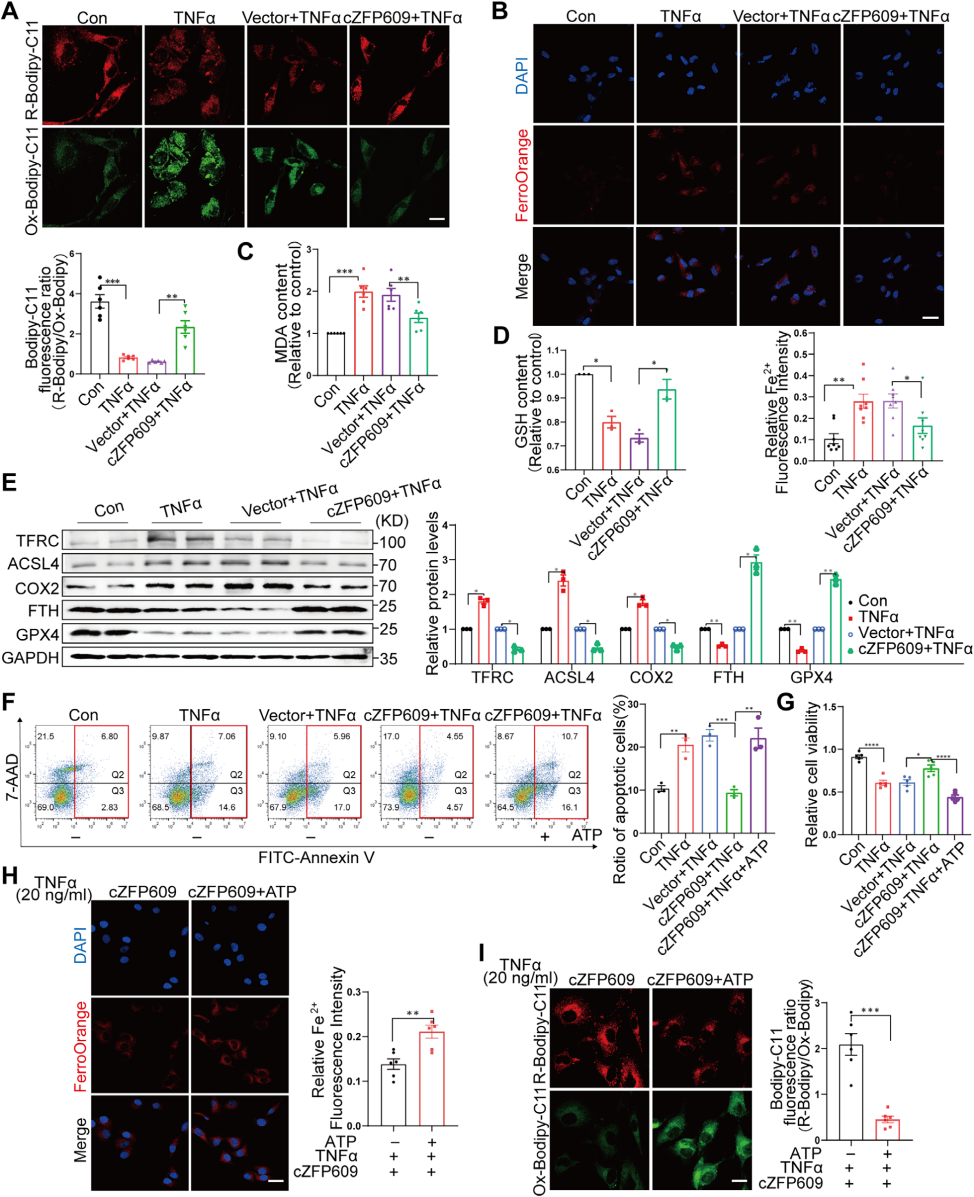
cZFP609 suppresses ferroptosis of chondrocytes. (A) Representative images of BODIPY (581/591) C11 staining in chondrocytes treated with the indicated conditions. The relative fluorescence intensity of R‐Bodipy/Ox‐Bodipy was quantified using Fiji images. Scale bar = 20 µm. (B) Representative images and relative quantification of FerroOrange staining. Scale bar = 40 µm. (C, D) MDA (C) and GSH (D) levels were detected in chondrocytes. (E) Western blot analysis of TFRC, ACSL4, COX2, FTH, and GPX4 proteins in chondrocytes. (F) Representative images from flow cytometry in chondrocytes. The total percentage of apoptosis (red square) is equal to the percentage of early apoptosis (Q2, Annexin V^+^7‐AAD^+^) plus the percentage of late apoptosis (Q3, Annexin V^+^7‐AAD^+^). (G) The cell viability of the chondrocytes was determined by CCK‐8 analysis. (H) Representative images and relative quantification of FerroOrange staining in chondrocytes treated with the indicated conditions. Scale bar = 40 µm. (I) Representative images of BODIPY (581/591) C11 staining and relative quantification. Scale bar = 20 µm. Data and images represent at least three independent experiments by Sidak's multiple comparisons test. *p < 0.05, **p < 0.01, ***p < 0.001, and ****p < 0.0001 versus the corresponding control.

The mitochondrion is a major organelle for reactive oxygen species (ROS) formation, and mitochondrial dysfunction participates in the development of ferroptosis [17]. As mentioned above, cZFP609 was essential for mitochondrial functions and homeostasis. Next, we investigated the effect of the mitochondrial function regulated by cZFP609 on ferroptosis. Given that lipid hydroperoxide accumulation and iron overload are the hallmarks of ferroptosis, we detected lipid peroxidation using C11 BODIPY 581/591, a fluorescent probe for membrane‐localized ROS. We showed that the overexpression of cZFP609 inhibited TNFα‐induced lipid peroxidation in chondrocytes. Indeed, upon TNFα treatment, cZFP609‐overexpressed cells had a decreased lipid peroxidation level (Figure 7A), diminished excessive iron (Figure 7B), and reduced malondialdehyde (MDA) (Figure 7C), accompanied by an increase in reduced glutathione (GSH) content (Figure 7D). Coincidentally, the expression of ferroptosis‐related proteins ACSL4 (acyl‐CoA synthetase long chain family member 4) and COX2 (prostaglandin‐endoperoxide synthase 2) was downregulated, and GPX4 (glutathione peroxidase 4) was upregulated in TNFα‐treated chondrocytes following overexpression of cZFP609 (Figure 7E). The excessive iron resulted from transferrin receptor (TFRC) accumulation, and ferritin H (FTH) chain reduction was significantly alleviated by cZFP609 overexpression (Figure 7E), suggesting a reduced ferroptosis. We used Erastin to inhibit GPX4 activity, and an increased ferroptosis was observed upon GPX4 inhibition, suggesting that cZFP609 suppressed ferroptosis via upregulation of GPX4 expression (Figure S5). Meanwhile, the apoptosis of chondrocytes was reduced by cZFP609 overexpression compared to TNFα‐treated cells, which was negated by ATP treatment (Figure 7F,G). Nevertheless, in the presence of ATP, TNFα‐induced ferroptosis was not abrogated by cZFP609 overexpression, which exhibited continuously increased excessive iron and lipid peroxidation (Figure 7H,I). Collectively, these results suggest that cZFP609 reduces ERMCs via inhibiting the depolymerization of BiP, contributing to mitochondrial function and chondrocyte survival.

### 2.8 Local Injection of cZFP609 Plasmid Retards OA Progression in Vivo

**FIGURE 8:**
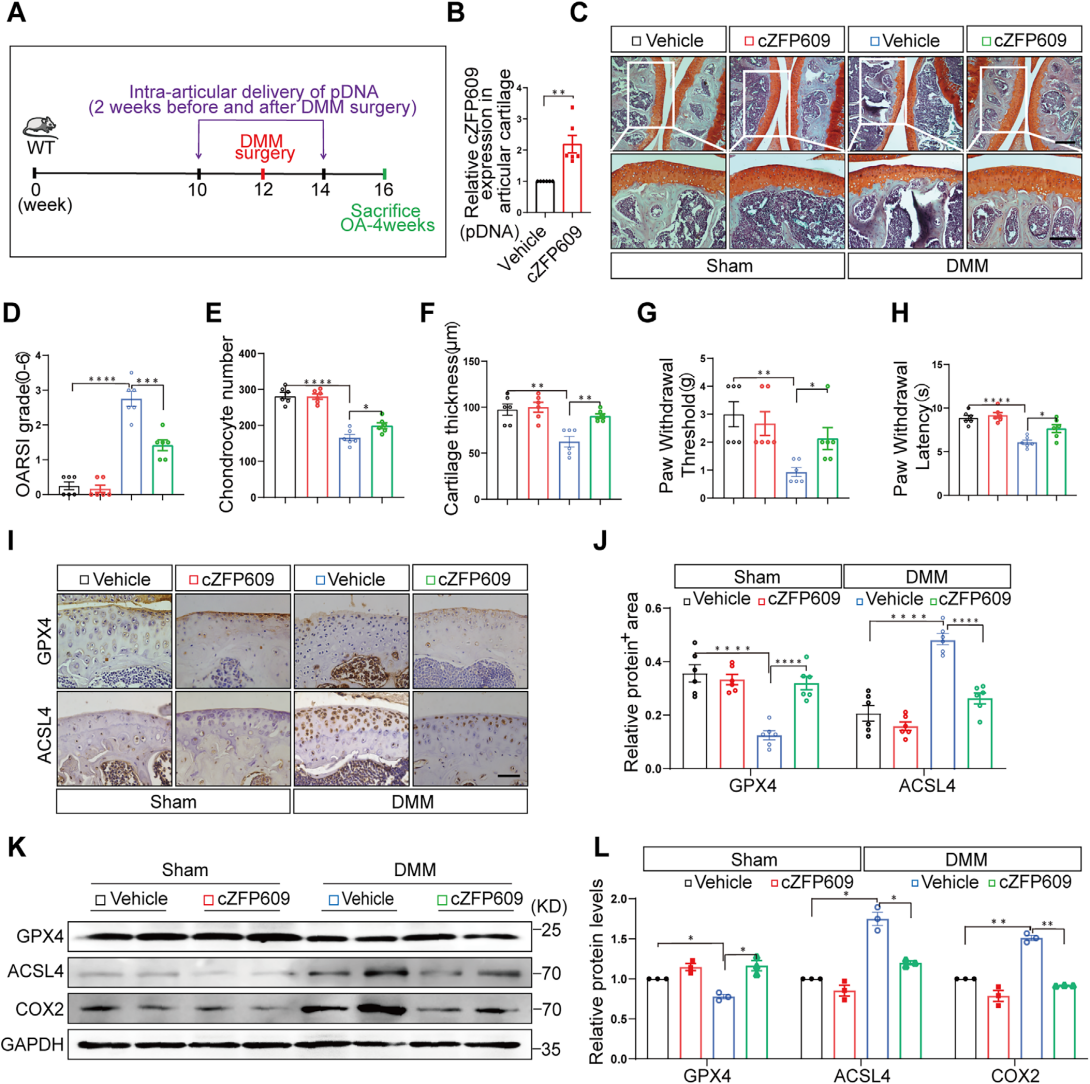
Intra‐articular injection of cZFP609 plasmid alleviates OA progression. (A) Schematic of the experimental timeline receiving sham or DMM surgery and intra‐articular cZFP609 plasmid DNA (pDNA) injection in wild‐type mice (*n* = 6). (B) The efficiency of cZFP609 was determined by RT‐qPCR in cartilage tissue at sacrifice. (C–F) Safranin‐O/fast green staining (C) and scoring of OA parameters, including OARSI grade (D), chondrocyte number (E), and cartilage thickness (F). Scale bar = 100 µm (*n* = 6). (G, H) PWT (G) and PWL (H) were used to evaluate mechanical pain sensitivity and hyperalgesia. (I, J) Immunohistochemical staining of GPX4 and ACSL4 proteins in the cartilage tissue (I) and quantitative analysis (J). Scale bar = 50 µm. (K, L) Western blot (K) and quantification analysis (L) of the protein levels of GPX4, ACSL4, and COX2 in the cartilage tissue. Data and images represent at least three independent experiments. Statistical analyses, paired *t*‐tests, and Tukey's multiple comparisons test. *p < 0.05, **p < 0.01, ***p < 0.001, and ****p < 0.0001 versus the corresponding control.

Finally, we investigated the effects of cZFP609 on OA by intra‐articular injection of cZFP609‐expressing plasmid in vivo. We treated mice as shown in the diagram (Figure 8A) and confirmed cZFP609 overexpression in the articular cartilage of mice receiving injection (Figure 8B, Figure S6). Administration of both empty vector control and cZFP609‐expressing plasmid did not affect the joint structure in the sham group (Figure 8C). The cartilage from the control OA mice had greater proteoglycan loss, roughness of the cartilage surface, and severe cartilage erosion, as indicated by elevated OARSI scores (Figure 8D). In contrast, treatment with cZFP609 plasmid attenuated the cartilage destruction, with cartilage preservation and reduced OARSI score. Accordingly, the chondrocyte number and cartilage thickness were increased (Figure 8E,F), accompanied by attenuated OA joint pain based on von Frey fibers and thermal radiometer assays following intra‐articular delivery of cZFP609 (Figure 8G,H). Consistently, administration of cZFP609 was able to reduce ferroptosis activity, as indicated by decreased ACSL4 and COX2 and increased GPX4 level in the OA cartilage (Figure 8I–L), suggesting efficacy in impeding OA progression by inhibiting ferroptosis.

## 3 Discussion

Emerging evidence has demonstrated that circRNAs are involved in the regulation of chondrocyte proliferation, autophagy, inflammation, apoptosis, and ECM metabolism in OA, and thereby delay or aggravate OA progression [18]. The current study demonstrated that vascular exosome cZFP609 protects against the degradation of articular cartilage through relieving chondrocyte ferroptosis in mouse OA models. The effects of cZFP609 on the chondrocytes confer joint health, and its loss could contribute to OA progression. We confirmed that cZFP609 stabilized the oligomeric form of BiP via the interaction between them in chondrocytes to minimize the ER stress and mitochondrial dysfunction induced by depolymerization of oligomeric BiP to the greatest extent possible under inflammatory conditions. Furthermore, cZFP609–BiP axis suppressed chondrocyte ferroptosis through abolishing deleterious ER–mitochondria communication driven by BiP‐related ERMCs. Finally, we assessed the potential therapeutic role of cZFP609 in OA in vivo. Consistent with observation in vitro, overexpression of cZFP609 by intra‐articular delivery was able to reduce ferroptosis in the OA cartilage and attenuated the cartilage degeneration, as indicated by increased chondrocyte number and cartilage thickness and reduced OARSI score, suggesting that cZFP609 is a potential protective factor for OA.

BiP function is regulated by reversible inactivating covalent modification and oligomerization/depolymerization equilibrium [19]. The modified and oligomeric BiP is its inactivating form [15]. In vivo, inactive BiP with substrate‐free is mainly oligomeric, while protein‐bound BiP is active and monomeric [20]. The active form of BiP in the ER is strictly regulated by post‐translational modification and oligomer formation [21]. Under unstressed conditions, BiP oligomers are tethered to ER stress sensor IRE1 [22]. The binding of ATP promotes the depolymerization of BiP oligomers and improves the stabilization of the monomeric form [23], which causes the dissociation of BiP from IRE1 and binds misfolded proteins to induce UPR [11]. We next conducted cZFP609 pull‐down proteomic analysis using mass spectrometry, identified BiP as a candidate, and validated that cZFP609 bound and stabilized the oligomeric BiP, which tethered BiP to IRE1α, and thereby reduced ER stress and inflammation.

Increasing lines of evidence suggest that mitochondria are closely connected to the ER via ERMCs, allowing them to communicate physically and biochemically [24]. Accordingly, ERMCs are involved in numerous fundamental metabolic processes [25], including phospholipid metabolism, the transfer of calcium and ROS between these two organelles, and the regulation of mitochondrial dynamics and bioenergetics [26]. BiP has been shown to form a complex with Sig‐1R (sigma‐1 ER receptor) and IP3R3 at the ERMCs, which prevents protein aggregation and transmits apoptotic Ca^2+^ signals to the mitochondria [11, 27]. Given that the conversion of BiP from an oligomeric to a monomeric state represents its activation, BiP‐related ERMC formation mediated deleterious ER–mitochondria communication, leading to mitochondrial dysfunction and ferroptosis, while cZFP609 blocked this process via a decrease in aberrant ERMC formation. Taken together, these results suggest that BiP‐related ERMC formation is an upstream key event of TNFα‐induced lipid peroxidation and ferroptosis of chondrocytes, and that cZFP609 targets BiP to block the biological sequence of the events via a decrease in aberrant ERMC formation by its binding and inhibiting BiP activation, which reduced ferroptosis. cZFP609 should be considered to be an RNA inhibitor of BiP activation and its downstream signaling events that are involved in chondrocyte degeneration in OA, including ER stress, mitochondrial dysfunction, and ferroptosis, and thereby enhances chondrocyte viability.

Iron is involved in a variety of vital functions, including DNA synthesis, oxygen transport, cellular respiration, and metabolic energy. Under physiologic conditions, cellular iron homeostasis is tightly regulated to ensure survival. Both iron deficiency and iron excess may affect the activity of iron‐dependent enzymes and the ability of oxygen transport and can also catalyze the formation of highly reactive hydroxyl radicals and ROS, leading to lipid peroxidation, genomic instability, DNA damage, and repair defects [28], which are associated with diabetes mellitus, nonalcoholic steatohepatitis, Parkinson's and Alzheimer's diseases, colon/breast cancer, and CVD [29]. Herein, we showed that the overexpression of cZFP609 abolished iron overload with decreased lipid peroxidation level and MDA content, which ultimately suppressed TNFα‐induced ferroptosis of chondrocytes. Based on the stability of circRNA, this intervention may have a long‐term protective effect on the articular cartilage of OA.

There are still some limitations in this study. First, we did not explore the roles of cZFP609 in human chondrocytes and cartilage explants. Thus, future studies should assess the role of cZFP609 in the chondrocytes isolated from patients with OA as a more representative comparison to our animal studies. Second, the effects of different doses of cZFP609 on OA treatment were not analyzed in detail. The optimal dosage and duration of cZFP609 administration need to be identified to achieve optimal therapeutic effect against OA progression. Lastly, the effect of cZFP609 on synovitis should be examined in treating OA in future studies to understand whether the effects of cZFP609 are tissue‐ and cell‐specific in this model.

In summary, this study demonstrates that cZFP609 inhibits chondrocyte ferroptosis and cartilage degradation by stabilizing the oligomeric form of BiP. Overexpression of cZFP609 effectively remedies BiP‐mediated aberrant ER–mitochondria communication in chondrocytes and retards OA progression. Collectively, our findings provide novel insights for the development of future OA therapies based on maintaining cartilage homeostasis, and cZFP609 is a promising RNA agent for OA treatment.

## 4 Materials and Methods

### 4.1 Animals and Ethics Statement

All animal manipulations were conducted with prior permission from the Animal Care and Use Committee of Hebei Medical University (JACUC‐Hebmu‐P2023003) and conformed to the Guide for the Care and Use of Laboratory Animals. Smooth muscle specific human sm*Sirt1*‐Tg mice were gifted by Dr. Chen H.Z., which were generated in a C57BL/6 background via the microinjection of human SIRT1 cDNA that was controlled by a minimal SM22α promoter (a region of the murine SM22α promoter that contains 445 base pairs of 5′‐flanking sequence). The expression of human SIRT1 mRNA and protein was validated in the arteries of the sm*Sirt1*‐Tg mice [4]. For routine identification of sm*Sirt1*‐Tg mice, PCR analysis was performed using the primers sense 5′‐CTTCAGGTCAAGGGATGGTAT‐3′ and antisense 5′‐GCGTGTCTATGTTCTGGGTAT‐3′, yielding a 233‐bp product from genomic DNA of the sm*Sirt1*‐Tg mice but not WT mice [4].

### 4.2 OA Model Induced by DMM and Intra‐Articular Injection of Plasmid DNA

DMM was performed to prepare an OA model of mice [30]. In brief, male, 4‐month‐old mice underwent the cut surgery of the meniscus ligament after being anesthetized with a mixture of oxygen and 1.125% isoflurane in the DMM group. The sham mice were used as a control. The intra‐articular injection of a plasmid vector containing cZFP609 or vehicle was performed before and after DMM surgery for 2 weeks. For each injection, mice were administered 20 µg of plasmid DNA.

### 4.3 Pain Assessment

The PWT and PWL were measured using von Frey fibers and a thermal radiometer [31]. The value of von Frey fibers was represented as PWT, and PWL represented the interval between the onset of thermal stimulation and the paw withdrawal. Each mouse was tested three times with an interval of at least 5 min.

### 4.4 Human Blood Sample Collection

The study was carried out according to the principles of Good Clinical Practice and the Declaration of Helsinki. Human blood samples were obtained from OA patients undergoing total knee arthroplasty at the Third Affiliated Hospital of Hebei Medical University (Shijiazhuang, China). The study recruited 20 patients with OA and 20 control subjects. Subjects with the following situations were excluded: undergoing surgery or trauma within 1 month prior to admission, severe infection, autoimmune disease, and malignancy.

### 4.5 Histology and Immunohistochemistry

After DMM surgery for 4 weeks, mice joints were cut out and, in sequence, fixed, decalcified, dehydrated, and embedded in paraffin. The sections (6‐µm‐thick) of the joint specimens were stained with Safranin O/fast Green dye. The severity of OA was assessed according to the Osteoarthritis Research Society International (OARSI) system [32] in a double‐blind manner. For immunohistochemistry, sections were incubated with antibodies against Col2ɑ (1:200, ab307674, Abcam), MMP13 (1:200, 18165‐1‐AP, Proteintech), Sox9 (1:100, sc‐166505, Santa Cruz), p53 (1:200, 10442‐1‐AP, Proteintech), GPX4 (1:100, A11243, ABclonal), and ACSL4 (1:200, 22401‐1‐AP, Proteintech). For visualization, 3,3′‐diaminobenzidine (DAB, ZLI‐9017, ZSGB‐BIO) was used. For exhibiting average OA development, six samples were quantified in each group.

### 4.6 Cell Culture and Treatment

Primary chondrocytes from articular cartilage of C57BL/6J mice (5‐day‐old) were harvested as previously described [30] and cultured in Dulbecco's modified Eagle's medium with 10% fetal bovine serum (FBS), 100 U/mL penicillin, and 100 µg/mL streptomycin. The cells grown to 90% confluence were passaged with 0.25% trypsin‐EDTA (Gibco, 25200‐072), and Passages 3–5 cells were used in the experiments. For stimulation, chondrocytes were treated with TNF‐α (20 ng/mL) following serum starvation for 24 h.

VSMCs were isolated from the aorta of mice and cultured in low glucose DMEM (Invitrogen, USA) supplemented with 10% FBS (Gibco), 100 U/mL penicillin, and 100 µg/mL streptomycin. The CM of VSMCs at Passages 3–5 was collected after incubation with DMEM containing 1% FBS for 24 h, centrifuged to remove the debris, and added to the chondrocyte cultures. For overexpression and knockdown of cZFP609, the plasmid or siRNA of cZFP609 was transfected into the cells. Human embryonic kidney (HEK) 293T cells with mycoplasma‐free (ATCC) that are identified by short tandem repeat (STR) analysis were maintained in high‐glucose DMEM containing 10% FBS.

### 4.7 Plasmid DNA or siRNA Transfection

The truncated cZFP609 expression plasmids were cloned into the pcD‐ciR vector (Youbao, Wuhan, China) [7] and verified by sequencing. The siRNA duplexes targeting mouse cZFP609 (si‐cZFP609), 5′‐GUCUGAAAAGCAAUGAUGUTT‐3′ and 5′‐ACAUCAUUGCUUUUCAGACTT‐3′, were obtained from GenePharma. Scrambled siRNA (si‐Con) 5′‐UUCUCCGAACGUGUCACGUTT‐3′ and 5′‐ACGUGACACGUUCGGAGAATT‐3′ served as a negative control. The chondrocytes grown to 50%–60% confluence were transfected with plasmid or siRNA using HighGene transfection reagent (ABclonal, Wuhan, China) according to the manufacturer's instructions. After transfection for 48 h, the expression of the target gene was quantified by qRT‐PCR.

Lentiviral vectors were generated by the transient cotransfection of HEK 293T cells with a three‐plasmid system [33]. The lentiviral vector system consists of pxPax2, pVSVG, and plasmids (PCDH‐GFP+PURO‐3xFlag, BiP PCDH‐GFP+PURO‐3xFlag, BiP‐NBD PCDH‐GFP+PURO‐3xFlag, or BiP‐Del PCDH‐GFP+PURO‐3xFlag, Youbao, Wuhan, China). Transfections were performed in 293T cells grown in 100‐mm dishes by optimized ratios of the constructs. The efficiency of transfection was determined by the expression of the green fluorescent protein reporter. The virus supernatant was collected for lentivirus concentration and purification, and then the purified lentivirus was used to infect 293T cells for subsequent experiments. Levels of BiP and its truncations (BiP‐NBD and BiP‐Del) were assayed by immunoblotting.

### 4.8 RNA‐Sequencing

Total RNAs were extracted from the chondrocytes and qualified by A260/A280 absorbance ratio detection before library construction. Paired‐end libraries were prepared using the ABclonal mRNA‐seq Lib Prep Kit (ABclonal, China) following the manufacturer's instructions and then sequenced on the Illumina NovaSeq 6000. Sequencing data were subjected to bioinformatics analysis using an in‐house pipeline from Shanghai Applied Protein Technology.

### 4.9 RNA Isolation and RT‐qPCR

After the indicated treatments, total RNAs of chondrocytes were extracted using Total RNA Extractor (B511311, Sangon Biotech, China) for qRT‐PCR. The cDNA was synthesized using the BeyoRT II First Strand cDNA Synthesis Kit (D7168M, Beyotime, China) and subjected to PCR using the BeyoFast SYBR Green qPCR Mix (2X, Low ROX) (D7262, Beyotime, China). The specificity of PCR products was confirmed by dissociation curve analysis and gel electrophoresis. All RNA expression was normalized to the amount of GAPDH using the 2^−ΔΔCT^ method.

### 4.10 Western Blot, Native‐PAGE, and Co‐Immunoprecipitation Analysis

The lysates of the tissues or cells were extracted by RIPA buffer (Beyotime, China) and quantified using the Quick Start Bradford protein assay (Bio‐Rad, CA, USA). Equal amounts of total protein (30–80 mg) were electrophoresed on SDS‐PAGE and transferred onto PVDF Membrane (Merck Millipore, Darmstadt, Germany). The membranes were blocked in 5% milk for 1 h at room temperature and then incubated with primary antibodies against MMP13 (1:1000, 18165‐1‐AP, Proteintech), Col2ɑ (1:1000, 28459‐1‐AP, Proteintech), Sox9 (1:200, sc‐166505, Santa Cruz), p53 (1:1000, 10442‐1‐AP, Proteintech), p16 (1:1000, A0262, ABclonal), p21(1:1000, HA500005, HUABIO), Sirt1 (1:500, A11267, ABclonal), IL‐1β (1:500, 16806‐1‐AP, Proteintech), BiP (1:1000, 11587‐1‐AP, Proteintech), IRE1α (1:1000, 27528‐1‐AP, Proteintech), IRE1α‐p (1:1000, AP0878, ABclonal), XBP‐1S (1:1000, 24868‐1‐AP, Proteintech), ATF4 (1:1000, 60035‐1‐Ig, Proteintech), IP3R (1:1000, A4436, ABclonal), TFRC (1:1000, A5865, ABclonal), GPX4 (1:1000, A11243, ABclonal), FTH (1:1000, 11682‐1‐AP, Proteintech), ACSL4 (1:1000, 22401‐1‐AP, Proteintech), COX2 (1:1000, ET1610‐23, HUABIO), and GAPDH (1:2000, AC036, ABclonal) at 4°C overnight. Membranes were probed with HRP‐conjugated secondary antibodies for 1 h at room temperature. The specific immunoreactive protein bands were visualized using the GE ImageQuant LAS 4000 detection system and analyzed using ImageJ Fiji (National Institutes of Health, USA). These experiments were replicated at least three times.

For the native‐PAGE, chondrocytes were lysed in native lysis buffer (20 mM Tris pH 7.4, 20 mM KCl, 5 mM MgCl_2_, 0.01% NP40) and used a native gel system (Seven Easy PAGE, SEVEN BIOTECH) for the analysis of BiP oligomers.

For co‐immunoprecipitation (co‐IP), the whole lysates were precleared with 20 µL protein A/Gagarose (Santa Cruz, USA) and then immunoprecipitated with 5 µg anti‐IRE1α (sc‐390960, Santa) antibodies at 4°C overnight. The immunoprecipitated protein was further analyzed using Western blot as described above.

### 4.11 Immunofluorescent Staining

Chondrocytes were fixed with 4% paraformaldehyde and permeabilized with 0.1% Triton X‐100 at room temperature for 15 min. Then, the cells blocked with 10% goat serum were incubated with antibodies against BiP (1:200, 11587‐1‐AP, Proteintech) and IP3R (1:100, A4436, ABclonal) at 4°C overnight and incubated with the fluorescein‐conjugated secondary antibody (1:200, Invitrogen) for 1 h. Nuclei were counterstained with DAPI Fluoromount‐G (0100‐20, SouthernBiotech). Images were acquired using a Leica microscope (Leica SP5, Switzerland) and digitized with the software program LAS AF Lite.

### 4.12 Measurement of Mitochondrial Morphology

Mitochondrial morphology was examined using MitoTracker Red FM (M7512, Invitrogen, USA) staining for 25 min at 37°C. Images of labeled mitochondria were captured by confocal microscopy (Leica SP5, Nussloch, Germany). The parameters of mitochondrial morphology were quantified using ImageJ macro tools. The experiment was repeatedly performed at least three times, and at least seven cells were analyzed per replicate.

### 4.13 Transmission Electron Microscopy

Chondrocytes were fixed in 2.5% glutaraldehyde at 4°C for 12 h and postfixed in 2% ferrocyanide‐reduced osmium tetroxide. Then, cells were stained with 1% uranyl acetate solution, dehydrated in progressively increasing concentrations of ethanol, passed through acetone, and embedded in Epon resin. Ultrathin sections were obtained, followed by counterstaining with 2% uranyl acetate and lead citrate. Images were acquired by a transmission electron microscope (120 kV; Tecnai G2 Spirit, FEI).

ERMCs were analyzed using ImageJ as previously described [34]. Additionally, the acquisition of electron microscope images was both randomly conducted and statistically analyzed in a double‐blind fashion. The ERMCs length was measured using the freehand line tool in ImageJ. The percentage of mitochondria surface area contacted with the ER was calculated by dividing ERMC's length by mitochondrial perimeter and multiplying by 100.

### 4.14 ATP Content Assay

ATP content was detected using an ATP assay kit (S0026, Beyotime, China). The cell lysate in ATP assay lysis buffer was centrifuged at 13,000 × *g* for 15 min at 4°C. The supernatant was collected and mixed with the ATP detection solution and tested using a multifunction microplate reader (SpectraMax i3, Thermo Fisher Scientific, USA). The experiments were independently performed at least three times.

### 4.15 Δψm Detection

After the indicated treatment, the cells were incubated with JC‐1 working solution (S2006, Beyotime, China) for 20 min and washed twice with 1 mL D‐Hank's. After the addition of 1 mL of imaging buffer solution, the cells were observed by a fluorescence microscope at wavelengths of 488 nm (green) and 594 nm (red).

### 4.16 Mitochondrial ROS Assay

For the mitochondrial ROS assay, the cells were stained with 5 µM MitoSOX Red fluorescence assay (M36008, Thermo Fisher Scientific, USA) at 37°C for 30 min and examined by Leica microscope. Data were analyzed using ImageJ Fiji software.

### 4.17 Lipid Peroxidation Assay

Lipid peroxidation was measured by 5 µM lipid peroxidation probe BODIPY 581/591 C11 (GC40165, GLPBIO, USA). Briefly, the medium was removed, and chondrocytes were incubated with the probe for 30 min at 37°C. After the staining, the cells were washed with PBS, sealed with DAPI Fluoromount‐G, and observed under a Leica SP5 laser confocal microscope. ImageJ Fiji software was used to analyze and detect lipid peroxidation.

In addition, MDA content was determined according to the manufacturer's instructions (S0131S, Beyotime, China), and the content of MDA in the initial sample was expressed as the amount of protein per unit weight.

### 4.18 Fe^2+^ Level Assay

Ferrous iron (Fe^2+^) was detected by the 1 µM Fe^2+^ probe FerroOrange (F374, Dojindo, Japan). The following steps are similar to that for the lipid peroxidation assay.

### 4.19 Fluorescence In Situ Hybridization

Chondrocytes were fixed in 4% paraformaldehyde for 10 min and permeabilized overnight in 70% ethanol. Then, the cells were rehydrated in hybridization buffer at 55°C for 4 h and then hydrated with 2 µg of fluorescence‐labeled specific probe of cZFP609 in hybridization buffer at 37°C for 12 h.

For FISH, after hybridization, chondrocytes were blocked with 10% BSA in PBS for 1 h, incubated with an antibody against BiP overnight at 4°C, and then incubated with a fluorescein‐conjugated secondary antibody (Invitrogen) at 37°C for 1 h. After washing three times with SSC, nuclei were counterstained with DAPI Fluoromount‐G. Images were acquired using a Confocal Laser Scanning Microscope System (Leica SP5, Nussloch, Germany) [7]. Relative quantitation for the FISH assay was conducted using the Fiji image macro tool. Co‐localization coefficients evaluated the co‐localization of fluorescence‐labeled specific probes of cZFP609 and BiP in chondrocytes based on pointed patterns from five different cells.

### 4.20 RIP Assay

RIP assay was conducted using antibodies against BiP or IgG (sc‐2027, Santa Cruz) in RIP buffer. The immunoprecipitated RNA‐antibody complexes were incubated with 50 µL Protein A/G PLUS‐Agarose beads (sc‐2003, Santa Cruz) for 2 h and washed with RIP buffer. Immunoprecipitated RNA was extracted using TRIzol (B511311, Sangon Biotech, China) and subjected to qRT‐PCR analysis using respective primers to demonstrate the presence of the binding products [35].

### 4.21 RNA Pull‐Down Assay

Chondrocytes were lysed in 500 µL lysis buffer and then incubated with 3 µg of biotinylated DNA oligonucleotide probes targeting cZFP609 at 4°C for 2 h. A total of 50 µL of BeyoMag Streptavidin Magnetic Beads (P2151, Beyotime, China) were added to the supernatant after it was centrifuged for 5 min at 12,000 g and further incubated at 4°C for 4 h. After incubation, the beads were washed three times using washing buffer. The bound proteins in the pull‐down materials were analyzed by Western blot [35].

### 4.22 Cell Viability Assay

Cell viability was assessed with the Cell Counting Kit‐8 assay (K1018, APExBIO Technology LLC, USA) according to the manufacturer's instructions.

### 4.23 Cell Apoptosis Assay

Apoptosis of chondrocytes was detected by Annexin‐V/7‐amino‐actinomycin D (7‐AAD) staining. Cells were collected with ice‐cold PBS by centrifugation, resuspended in 100 µL binding buffer, and stained with 2.5 µL FITC Annexin V (556547, BD Biosciences, USA) and 2.5 µL 7‐AAD (RM02985, ABclonal, China) for 15 min at 37°C. Cells were analyzed using a FACS Canto cytometer (Agilent NovoCyte Quanteon, China), and early and late cell death was evaluated on FITC fluorescence (Annexin V) versus PerCP (7‐AAD) plots by FlowJo Software.

### 4.24 Statistics

Statistical analyses were conducted with the SPSS 21.0 software. The data are presented as means ± SEM. Two groups were compared by Student's *t*‐tests. Differences among groups were analyzed with one‐way analysis of variance (ANOVA). *p* values below 0.05 were considered significant. Biologically independent sample numbers were shown in figures and figure legends. Data and images represent at least three independent experiments.

## Author Contributions

Y.S., Y.G., F.Z., J.‐L.L., J.S., and W.‐D.Z. conducted experiments and analyzed data. J.S. and S.D. prepared an OA model of mice. S.‐Y.C., H.‐B.J., D.‐D.Z., and P.K. assisted in the experiments. Y.S. analyzed data and generated the illustrations. H.L. and M.H. supervised the project and wrote the manuscript. All authors have read and approved the final manuscript.

## Ethics Statement

This research complied with the guidelines of the Chinese Committee on Animal Welfare, and all animal‐related procedures were performed in strict accordance with the approved protocol by the Hebei Medical University Animal Experimentation Ethics Committee (permit no. IACUC‐Hebmu‐P2023003). Regarding the human component, ethical approval was granted by the Hebei Medical University's Medical Ethics Committee (permit no. 2023‐003). Informed consent was obtained from all participants for blood collection and subsequent analysis. This research adhered to the Declaration of Helsinki (2013) and ARRIVE Guidelines.

## Conflicts of Interest

The authors declare no conflicts of interest.

## Supporting information




**Supplement table S1**. Characteristics of the study participants.
FIGURE S1. The genotype identification of sm*Sirt1*‐Tg mice. Genomic DNA of the sm*Sirt1*‐Tg mice was analyzed by PCR and yielded a 233‐bp product. Wild‐type (Lanes 1–6), sm*Sirt1*‐Tg (Lanes 7–12). The markers from top to bottom are 2000, 1000, 750, 500, 250 and 100 bp.
FIGURE S2. The efficiency of cZFP609 knockdown and overexpression.
(A and B) The expression of cZFP609 in sm*Sirt1*‐Tg VSMCs (A) and their conditional media (B) after knockdown of cZFP609. (C) The overexpression of cZFP609 in chondrocytes.
FIGURE S3. The expression of Col2ɑ mRNA in chondrocytes of passages 1‐7 cultured in vitro.
FIGURE S4. Heat map analysis of differentially expressed genes in chondrocytes with overexpression of cZFP609.
FIGURE S5. The expression of GPX4, ACSL4 and COX2 in chondrocytes.
(A) Western blot of GPX4, ACSL4 and COX2 expression in chondrocytes treated with Erastin following overexpression of cZFP609. (B) The corresponding quantitative analysis.
FIGURE S6. The expression of cZFP609 in the joint tissues of mice after intra‐articular injection of cZFP609 expression plasmid.

## Data Availability

The datasets utilized and analyzed in this research are accessible through the principal investigator upon request for legitimate purposes.
