# Supplementary material for: cZFP609 Tethering BiP Alleviates Cartilage Degradation in Osteoarthritis via Remedying Aberrant ER‐Mitochondrial Contacts
Source: MedComm (2020). 2025 Oct 13;6(10):e70405. doi: 10.1002/mco2.70405 (PMC12516089; doi:10.1002/mco2.70405)
Supplement: Supplementary file 1 — Supplement table S1. Characteristics of the study participants. FIGURE S1. The genotype identification of smSirt1‐Tg mice. Genomic DNA of the smSirt1‐Tg mice was analyzed by PCR and yielded a 233‐bp product. Wild‐type (Lanes 1–6), smSirt1‐Tg (Lanes 7–12). The markers from top to bottom are 2000, 1000, 750, 500, 250 and 100 bp. FIGURE S2. The efficiency of cZFP609 knockdown and overexpression. (A and B) The expression of cZFP609 in smSirt1‐Tg VSMCs (A) and their conditional media (B) after knockdown of cZFP609. (C) The overexpression of cZFP609 in chondrocytes. FIGURE S3. The expression of Col2ɑ mRNA in chondrocytes of passages 1‐7 cultured in vitro. FIGURE S4. Heat map analysis of differentially expressed genes in chondrocytes with overexpression of cZFP609. FIGURE S5. The expression of GPX4, ACSL4 and COX2 in chondrocytes. (A) Western blot of GPX4, ACSL4 and COX2 expression in chondrocytes treated with Erastin following overexpression of cZFP609. (B) The corresponding quantitative analysis. FIGURE S6. The expression of cZFP609 in the joint tissues of mice after intra‐articular injection of cZFP609 expression plasmid. [file MCO2-6-e70405-s001.docx]

**cZFP609 tethering BiP alleviates cartilage degradation in osteoarthritis via remedying aberrant ER-mitochondrial contacts**

Yu Song^1^, Jun-Long Luo^2^, Fan Zhang^1^, Jie Shi^1^, Shuai Du^2^, Hai-Bin Jiang^1^, Wen-Di Zhang^1^, Si-Ying Chen^1^, Dan-Dan Zhang^1^, Peng Kong^1^, Yuan Gao^1^*, Mei Han^1^*, Han Li^2^*

^1^ Department of Biochemistry and Molecular Biology, College of Basic Medicine, Key Laboratory of Neural and Vascular Biology of Ministry of Education, Key Laboratory of Vascular Biology of Hebei Province, Hebei Medical University, Shijiazhuang, China.

^2^ Department of Orthopaedic Surgery, Institute of Biomechanical Science and Biomechanical Key Laboratory of Hebei Province, Third Hospital of Hebei Medical University, Shijiazhuang, China.

*Corresponding authors: lihan1984@hebmu.edu.cn (H.L.), hanmei@hebmu.edu.cn (M.H.), [gy@hebmu.edu.cn](mailto:gy@hebmu.edu.cn) (Y.G.).

**Running title:** cZFP609-BiP axis inhibits cartilage degradation

**Supplement table S1. Characteristics of the study participants**

|  | All participants | Normal | OA | *P*-value |
| --- | --- | --- | --- | --- |
| Age | 61.7±7.4 | 60.9±8.8 | 62.5±5.7 | 0.4862 |
| Gender (male:female) | 23:17 | 11:9 | 12:8 |  |
| Height (cm) | 164.3±7.7 | 165.7±8.5 | 162.9±6.7 | 0.2420 |
| Weight (Kg) | 72.4±11.6 | 70.8±14.5 | 74.1±7.7 | 0.3871 |
| BMI | 26.9±3.6 | 25.7±3.5 | 28.2±3.4 | 0.0260 |
| SBP (mmHg) | 135.9±17.5 | 134.9±21.3 | 136.9±13.1 | 0.7222 |
| DBP (mmHg) | 85.1±12.7 | 81.8±15.4 | 88.5±8.5 | 0.0969 |
| TG (mmol/L) | 1.5±1.0 | 1.6±1.3 | 1.5±0.5 | 0.6550 |
| TC (mmol/L) | 4.9±1.0 | 5.0±1.3 | 4.8±0.7 | 0.5543 |
| HDL-C (mmol/L) | 1.3±0.3 | 1.5±0.4 | 1.2±0.3 | 0.0095 |
| LDL-C (mmol/L) | 3.0±0.9 | 2.9+1.1 | 3.2±0.6 | 0.3298 |
| Carotid Plaque | 60% | 65% | 50% |  |
| Hypertension | 55% | 55% | 55% |  |

Data are expressed as mean±SED, Ratio of number or n (%). *P*-values represent the comparison between Non-OA (Normal) and OA patients.

BMI, body mass index; OA, osteoarthritis; SBP, systolic blood pressure; DBP, diastolic blood pressure; TC, total cholesterol; TG, triglyceride; HDL-C, high-density lipoprotein cholesterol; LDL-C, low-density lipoprotein cholesterol.


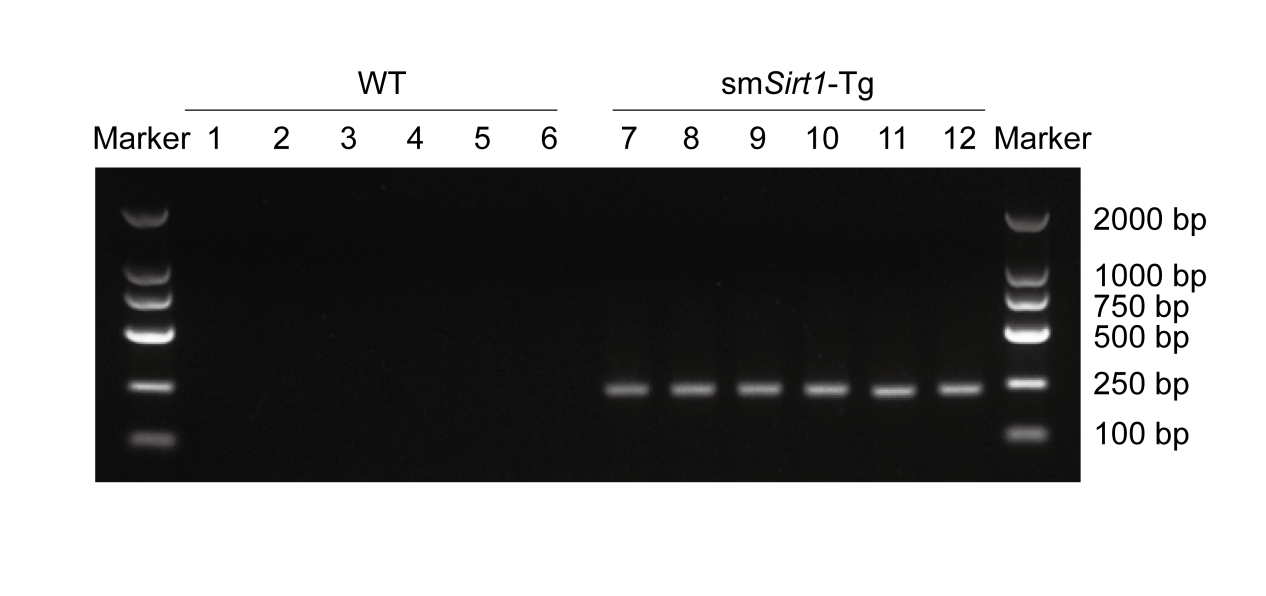


**FIGURE S1. The genotype identification of sm*Sirt1*-Tg mice.**

Genomic DNA of the sm*Sirt1*-Tg mice was analyzed by PCR and yielded a 233-bp product. Wild-type (lanes 1-6), sm*Sirt1*-Tg (lanes 7-12). The markers from top to bottom are 2000, 1000, 750, 500, 250 and 100 bp.


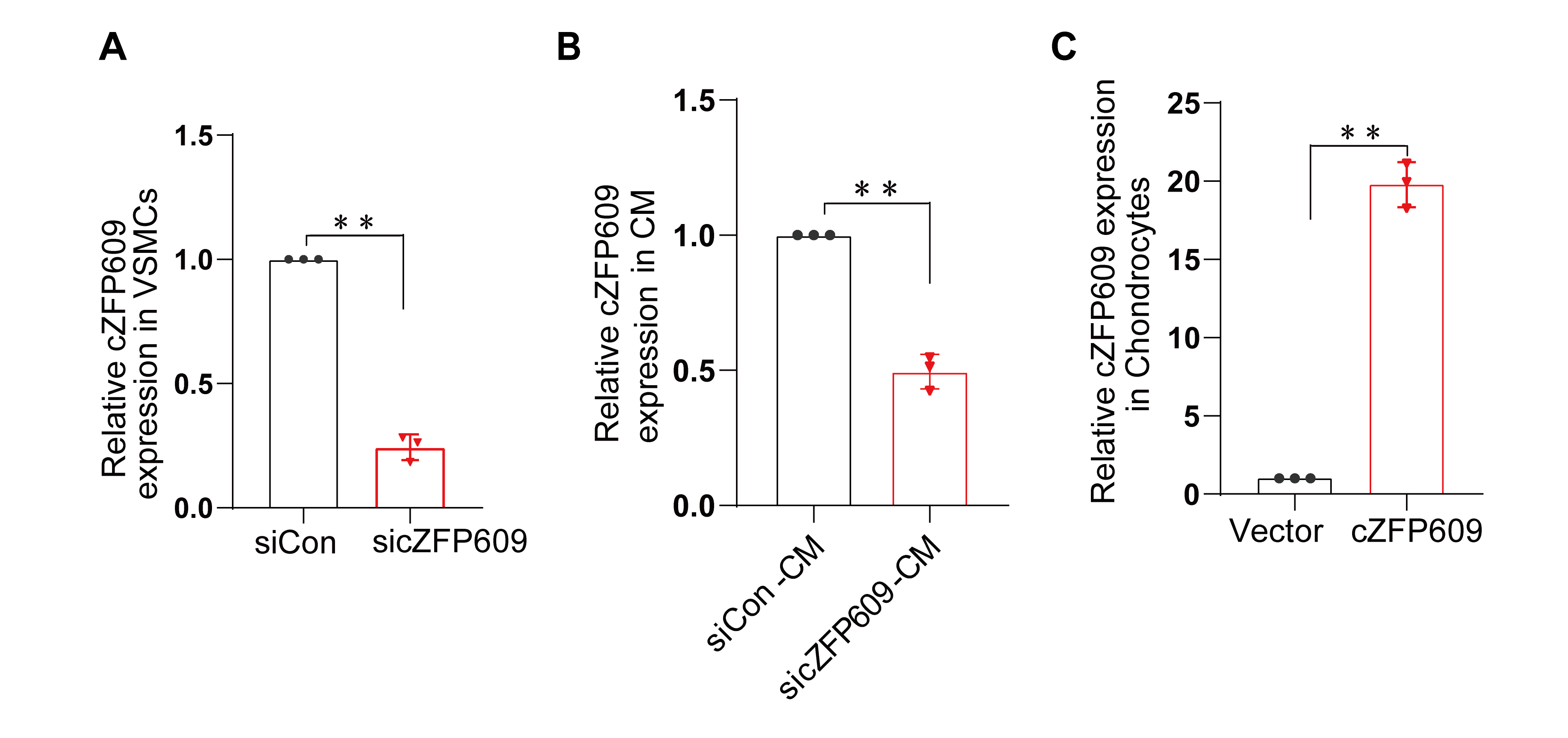


**FIGURE S2. The efficiency of cZFP609 knockdown and overexpression.**

(A and B) The expression of cZFP609 in sm*Sirt1*-Tg VSMCs (A) and their conditional media (B) after knockdown of cZFP609. (C) The overexpression of cZFP609 in chondrocytes.

**
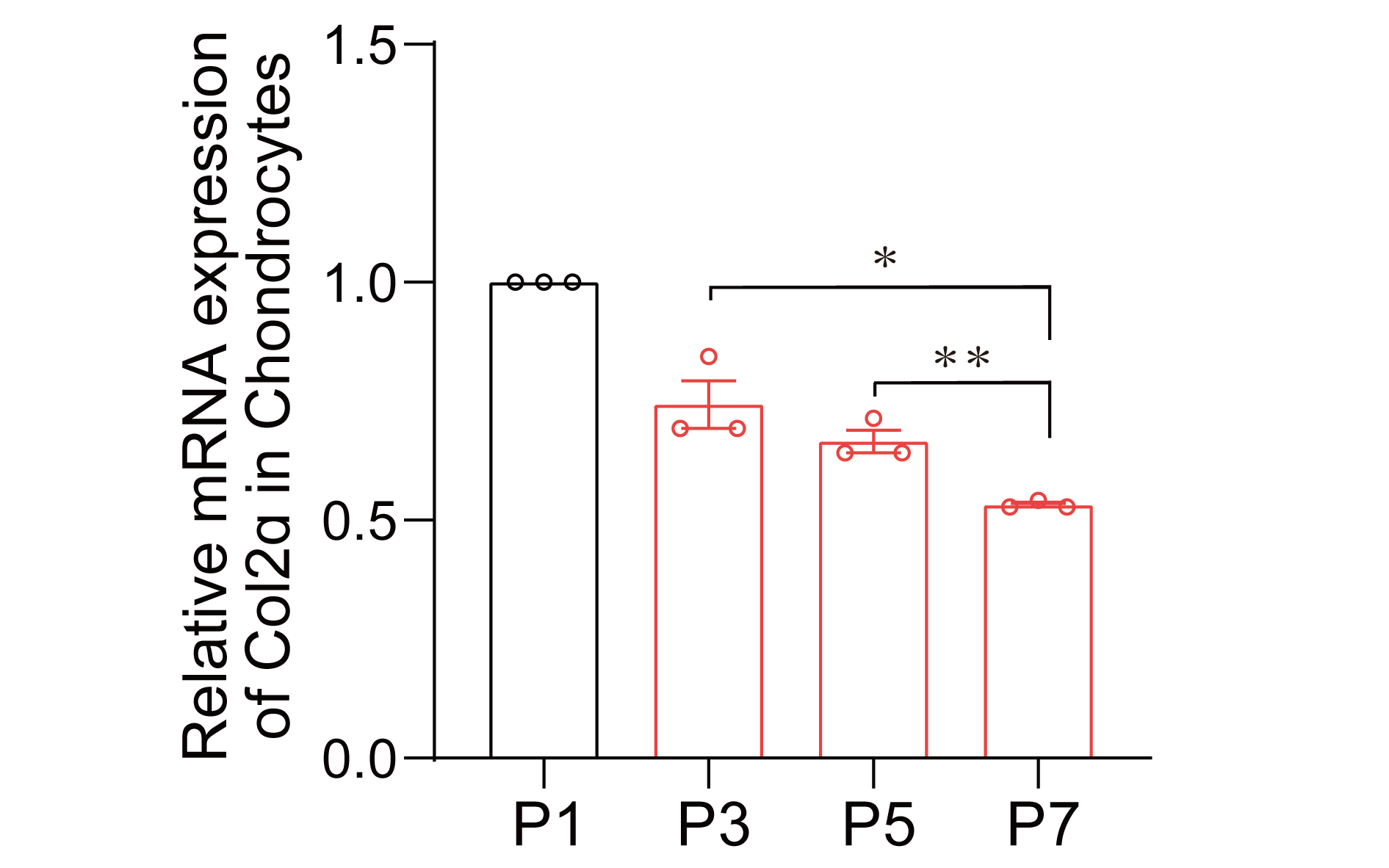
**

**FIGURE S3. The expression of Col2ɑ mRNA in chondrocytes of passages 1-7 cultured *in vitro*.**

**
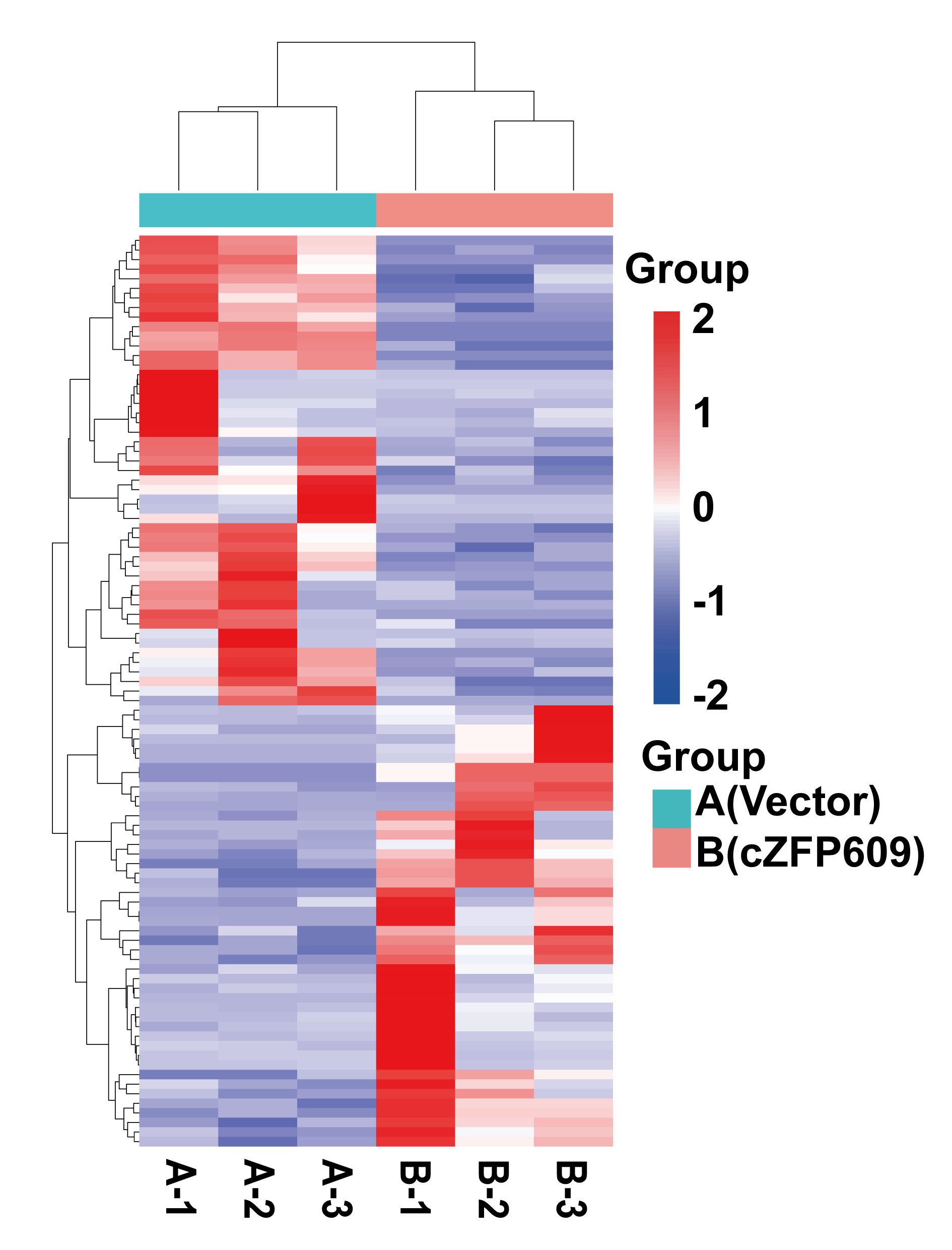
**

**FIGURE S4. Heat map analysis of differentially expressed genes in chondrocytes with overexpression of cZFP609.**

**
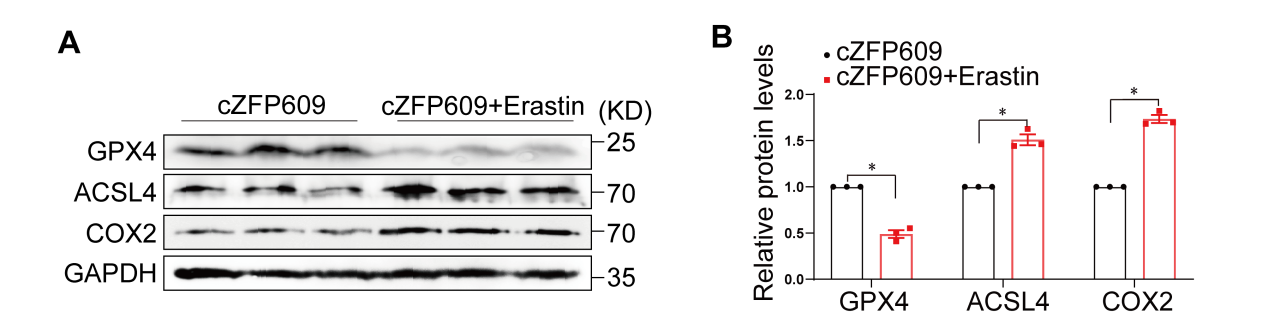
**

**FIGURE S5. The expression of GPX4, ACSL4 and COX2 in chondrocytes.**

(A) Western blot of GPX4, ACSL4 and COX2 expression in chondrocytes treated with Erastin following overexpression of cZFP609. (B) The corresponding quantitative analysis.

**
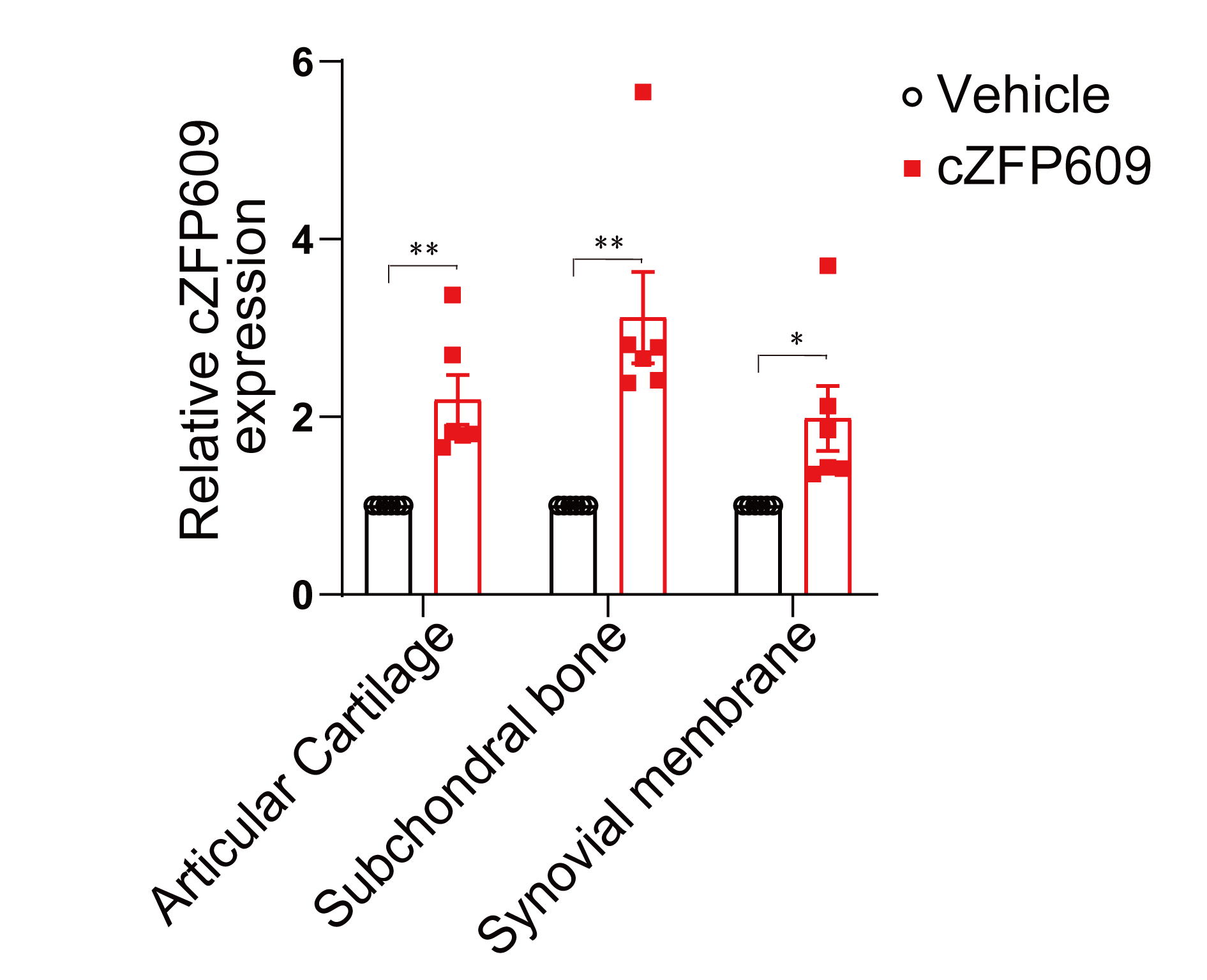
**

**FIGURE S6. The expression of cZFP609 in the joint tissues of mice after intra-articular injection of cZFP609 expression plasmid.**
